# Rescue-like behavior in a bystander mouse toward anesthetized conspecifics promotes arousal via a tongue-brain connection

**DOI:** 10.1126/sciadv.adq3874

**Published:** 2025-01-22

**Authors:** Peng Cao, Ying Liu, Ziyun Ni, Mingjun Zhang, Hong-Rui Wei, An Liu, Jin-Rong Guo, Yumeng Yang, Zheng Xu, Yuyu Guo, Zhi Zhang, Wenjuan Tao, Likui Wang

**Affiliations:** ^1^ Department of Anesthesiology, The First Affiliated Hospital of USTC, Hefei National Laboratory for Physical Sciences at the Microscale, Division of Life Sciences and Medicine, University of Science and Technology of China, Hefei 230026, China.; ^2^ Department of Physiology, School of Basic Medical Sciences, Anhui Medical University, Hefei 230022, China.; ^3^ NMPA Key Laboratory for Research and Evaluation of Narcotic and Psychotropic Drugs, Xuzhou Medical University, Xuzhou 221004, China.; ^4^ Department of Pain Medicine, The First Affiliated Hospital of Anhui Medical University, Hefei 230022, China.; ^5^ College & Hospital of Stomatology, Key Laboratory of Oral Diseases Research of Anhui Province, Anhui Medical University, Hefei 230022, China.

## Abstract

Prosocial behaviors are advantageous to social species, but the neural mechanism(s) through which others receive benefit remain unknown. Here, we found that bystander mice display rescue-like behavior (tongue dragging) toward anesthetized cagemates and found that this tongue dragging promotes arousal from anesthesia through a direct tongue-brain circuit. We found that a direct circuit from the tongue → glutamatergic neurons in the mesencephalic trigeminal nucleus (MTN^Glu^) → noradrenergic neurons in the locus coeruleus (LC^NE^) drives rapid arousal in the anesthetized mice that receive the rescue-like behavior from bystanders. Artificial inhibition of this circuit abolishes the rapid arousal effect induced by the rescue-like behavior. Further, we revealed that glutamatergic neurons in the paraventricular nucleus of the thalamus (PVT^Glu^) that project to the nucleus accumbens shell (NAcSh) mediate the rescue-like behavior. These findings reveal a tongue-brain connection underlying the rapid arousal effects induced by rescue-like behavior and the circuit basis governing this specific form of prosocial behavior.

## Teaser

Bystander mice exhibit rescue-like behavior, promoting arousal in anesthetized mice via a direct tongue-brain circuit.

## INTRODUCTION

Prosocial behaviors are essential for survival and thriving in some species (*1*–*4*). Previous studies have shown that both humans and rodents exhibit a range of prosocial behaviors such as consolation, helping, cooperating, and sharing food to improve their emotional state or to assist other individuals in escaping from distressing situations (*5*–*10*). While empathy may play a role in motivating prosocial behavior, other motivations are possible. Helping or rescuing behaviors, for instance, may involve deliberate and targeted actions to address the specific needs of a distressed conspecific, which could be driven by a range of factors, including the helper’s own distress when unable to escape a conspecific’s precarious situation (*7*, *8*, *11*). For example, previous studies have revealed helping behaviors among bystander mice toward their restrained counterparts, reminiscent of how humans aid each other in distress (*12*–*14*).

In both humans and experimental animals, anesthesia is common, and painful stimuli can promote arousal from anesthesia (*15*–*17*). In traditional Chinese medicine for emergency treatment, a pinching or acupuncture at the Renzhong acupoint (GV 26, above the upper lip on the midline) on the face during fainting or general anesthesia can elicit an awakening effect (*18*, *19*). In a human social context, fainting is a salient social signal, prompting helpful responses such as physical assistance from others to awaken the individual (*20*). However, it remains unclear whether mice can engage in similar prosocial behaviors to promote arousal in anesthetized companions.

The nucleus accumbens (NAc) has been previously shown to function in mediating goal-directed approach behaviors, including approach toward social targets (*21*). Inhibition of NAc-related projections and inhibition of oxytocin receptors have been shown to result in reduced social exploration in normal mice (*22*, *23*), while activation of serotonin receptors within the NAc enhances sociability in an autism mouse model (*24*). A recent study demonstrated that distinct ensembles of NAc neurons are activated during approach toward partners and novel conspecifics in prairie voles (*25*). Dopaminoceptive medium spiny neurons (MSNs) comprise the main neuronal population (~90%) in the NAc, which were divided into two subpopulations: D1- (D1-MSNs) or D2-dopamine receptor-expressing MSNs (D2-MSNs) (*26*). These two MSN subpopulation have different outputs and play distinct, often opposing, roles in motivated behaviors: Activation of D1-MSNs is rewarding, while activation of D2-MSNs is aversive (*27*). However, whether MSNs contribute to the modulation of helping behaviors during prosocial interactions remains unclear.

In the present study, we established a model of prosocial interactions for bystander mice towards anesthetized cagemates and found that bystander mice engage in rescue-like behavior (“tongue dragging”) to promote arousal of anesthetized mice. We then dissected the functional organization of the circuit that drives the rescue-like behavior by bystander mice, comprising glutamatergic neurons in the paraventricular nucleus of the thalamus (PVT^Glu^) → parvalbumin (PV)–expressing interneurons (INs) in the NAc shell (NAcSh^PV^), as well as the circuit underlying the observed arousal in anesthetized mice, comprising tongue → glutamatergic neurons in the mesencephalic trigeminal nucleus (MTN^Glu^) → noradrenergic neurons in the locus coeruleus (LC^NE^). Chemogenetic activation of the PVT^Glu^ → NAcSh^PV^ circuit increases the rescue-like behavior and chemogenetic activation of the tongue → MTN^Glu^ → LC^NE^ circuit promotes arousal in anesthetized mice. Conversely, artificial inhibition of each neural circuit separately reduces the rescue-like behavior and abolishes its effect of promoting arousal in anesthetized mice. Moreover, we found that NAcSh^D1-MSN^ but not NAcSh^D2-MSN^ neurons regulate rescue-like behavior in bystander mice. These findings reveal how bystanders respond to anesthetized cagemates and illustrate the circuit basis governing this specific form of prosocial behavior.

## RESULTS

### Bystander mice exert rescue-like behavior that promotes arousal in anesthetized mice

**Fig. 1.**
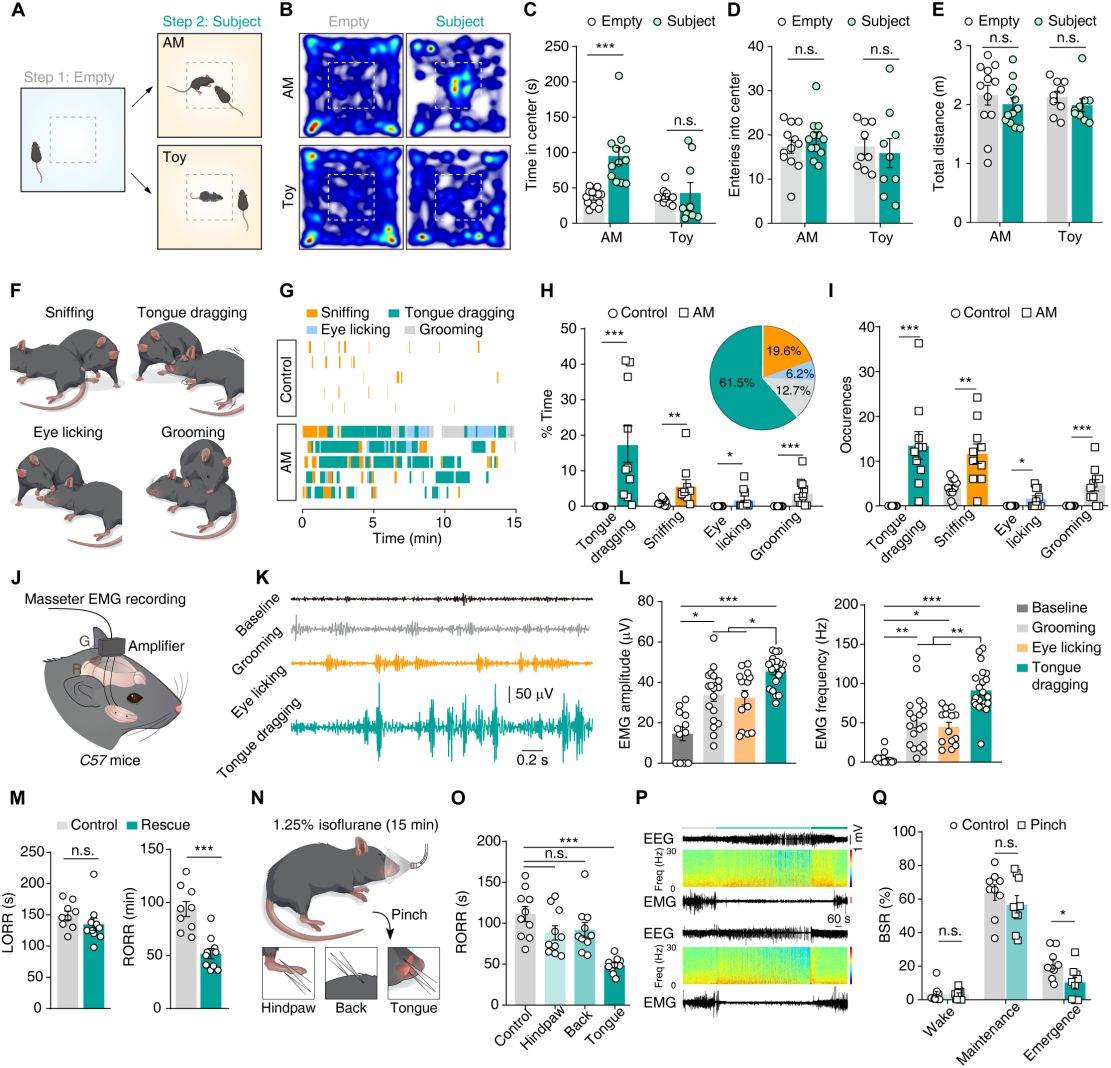
Bystander mice exhibit targeted rescue-like behavior that promotes arousal in anesthetized mice. (**A**) Experimental paradigm for bystander mouse interacts with anesthetized mouse (AM). (**B** to **E**) Representative heatmaps (B) and summary data [(C) to (E)] of bystander mice paired with AM or toy mice in the open field test. *n* (AM) = 12, *n* (toy) = 9. (**F** and **G**) Schematic diagram (F) and example raster (G) showing the various behaviors observed in bystander mice with AM or saline-treated controls. (**H** and **I**) Total duration percentage (H) and occurrences (I) for various behaviors of bystander mice toward saline-treated controls or AM. Pie charts indicate the proportion of behavioral occurrence in (H). *n* = 10. (**J** and **K**) Schematic diagram (J) and example EMG traces (K) showing EMG recording from masseter muscles of bystander mice. (**L**) Quantitation of EMG amplitude and frequency. *n* (baseline, grooming, eye licking, and tongue dragging) = 12, 19, 14, and 21 trails from five mice, respectively. (**M**) Time of anesthetic induction (LORR, loss of righting reflex) and recovery (RORR, recovery of righting reflex) after intraperitoneal injection of chloral hydrate in AM in the presence (rescue) or absence (control) of bystander mice. *n* (control) = 9, *n* (rescue) = 10. (**N**) Schematic for the application of pinch stimuli to the hindpaw, back, or tongue of isoflurane-AM. (**O**) Summary data for the RORR from the isoflurane-AM. *n* = 10. (**P**) Examples of EEG and EMG data from isoflurane-AM treated with (pinch) or without tongue pinching (control) during the emergence period. Continuous EEG spectrograms are plotted underneath the raw EEG traces. (**Q**) Summary data for the BSR in the wake, maintenance, and emergence periods. *n* = 9. Data are presented as the means ± SEMs. *P < 0.05, **P < 0.01, and ***P < 0.001; n.s., not significant. Details of the statistical analyses are presented in table S1.

Inspired by previous studies reporting that other mice will open the lid of a restrainer tube to free their cagemates (*14*), we here developed an experiment to assess whether bystander mice (i.e., naïve to anesthesia) express interest in the presence of anesthetized cagemates. Briefly, we quantitatively assessed the movements of bystander mice upon placing either a decoy (toy) mouse or an anesthetized cagemate mouse into the experimental cage (Fig. 1A). We found that the bystander mice spent significantly more time in the central area of the open field apparatus when anesthetized mice were present; no preference for the central area was evident for the toy mice (Fig. 1, B to D). This place preference, when considered alongside our finding of no difference in total movement distance (Fig. 1E), supports the notion that bystander mice express interest in anesthetized mice.

We subsequently examined the interactions between the bystander and anesthetized mice in greater detail, ultimately discerning four distinct behaviors exerted by the bystanders toward their anesthetized cagemates: sniffing, grooming, eye licking, and a “tongue dragging” behavior in which the bystander mouse initially uses its teeth to grasp the tongue of the anesthetized mouse until it protrudes from the mouth, after which the tongue is again grasped by the teeth of the bystander mouse and “dragged” repeatedly (Fig. 1, F and G, and movies S1 and S2). Notably, the duration and number of occurrences of tongue dragging were significantly higher than the other three behaviors (Fig. 1, H and I). Given that bystander mice may exhibit different prosocial behaviors toward familiar and unfamiliar mice, we then examined the interactions between the bystander mice and anesthetized cagemates or anesthetized stranger mice and found no notable difference in these prosocial behaviors (fig. S1). Similarly, female bystander mice also exhibit tongue-dragging behaviors toward anesthetized mice (fig. S2, A to C). However, we noted that female bystander mice exhibited significantly fewer tongue-dragging behaviors toward stranger anesthetized mice (fig. S2, D to G). These findings reveal a sex-specific familiarity effect, wherein females but not males are less likely to engage in this tongue-dragging behavior toward strangers. Male mice were used in the remaining experiments throughout the study.

We explored possible overlap in the tongue-dragging with face-grooming behavior in bystander mice, specifically by recording electromyograms (EMGs) from the masseter muscles of freely moving mice (*28*). We found that masseter EMG amplitude and frequency were each significantly increased in bystander mice when they engaged in tongue dragging on anesthetized mice, as compared to the baseline levels observed when bystander mice were freely exploring the environment (Fig. 1, J to L). Notably, the EMG amplitude and frequency were higher in bystander mice when engaging in tongue dragging than when engaging in face grooming and eye licking (Fig. 1, K and L). These results indicate both that bystander mice do exhibit tongue-dragging behavior toward anesthetized mice and that this is distinct from face-grooming behavior.

Because the anesthetized mice exhibit leg shaking and body trembling when bystander mice drag their tongues (movies S1 and S2), we speculate that this behavior possibly exerts an arousal-promoting effect. Intriguingly, we noted that the anesthetized mice recovered faster from general anesthesia after tongue dragging by bystander mice than did control mice with no bystander mice present, as evidenced by a significant reduction in the recovery of righting reflex (RORR) time in the tongue-dragging mice compared with that in the controls, although there was no notable difference in the loss of righting reflex (LORR) between these two groups (Fig. 1M). Thus, tongue dragging by bystander mice can be understood as a rescue-like prosocial behavior, as it is elicited by apparently distressed cagemates and results in a significantly faster recovery of the anesthetized animals. Notably, we found that bystander mice exhibit rescue-like behaviors only toward living, anesthetized mice (i.e., not cadavers) (fig. S3).

Our detection of this rescue-like behavior and its impact on cagemates motivated us to conduct a series of experiments to determine how tongue dragging ultimately results in the observed promotion of arousal from anesthesia (i.e., the reduction in post-anesthesia emergence time). We established isoflurane anesthesia protocols of 2.5% for 5 min and 1.25% for 15 min (*29*). We administered a pinch stimulus to the hindpaw, back skin, or tongue of anesthetized mice during the emergence period and found that only the tongue stimulation resulted in a significant shortening of the RORR time compared to control mice (Fig. 1, N and O, and fig. S4, A to D).

The burst suppression ratio (BSR) is a characteristic phenomenon used in studies of deep anesthesia (*29*). We conducted electroencephalogram (EEG) and EMG recordings to monitor brain activity in both control and pinched mice using both aforementioned anesthesia regimes (Fig. 1P and fig. S4E). Analysis of the EEG spectrum in the waking and maintenance periods revealed no difference in the BSR, whereas the BSR was significantly reduced in tongue-pinched mice compared to control mice during the emergence period (Fig. 1Q and fig. S4F). Therefore, administration of tongue stimulation during the emergence period promotes arousal of anesthetized mice.

### MTN^Glu^ neurons directly project to the tongue

We next investigated which nuclei in the brain are involved in this phenomenon, wherein a rescue-like behavior (tongue dragging) by bystanders promotes arousal in anesthetized mice. A retrograde monosynaptic tracing dye, Fluoro-Gold (FG), was injected into the tongue of C57 mice to explore the brain origin of the tongue nerves (fig. S5A). One week later, FG^+^ signals were observed in the keratin layer and epithelial tissue of the tongue, which were labeled with a marker for peripheral nerve fibers (fig. S5B). FG^+^ neurons were also observed in the trigeminal ganglion and the MTN (fig. S5, C to E), which is the only known nucleus in the brain that contains primary afferent sensory neurons that are related to processing oral sensory motor information (*30*, *31*). The MTN and LC are positioned in close proximity in the mouse brain, and immunofluorescence staining for the catecholaminergic marker tyrosine hydroxylase (TH) indicated that FG^+^ neurons were located in the MTN but not the LC (fig. S5D). These results support the notion that MTN neurons innervate the tongue.

**Fig. 2.**
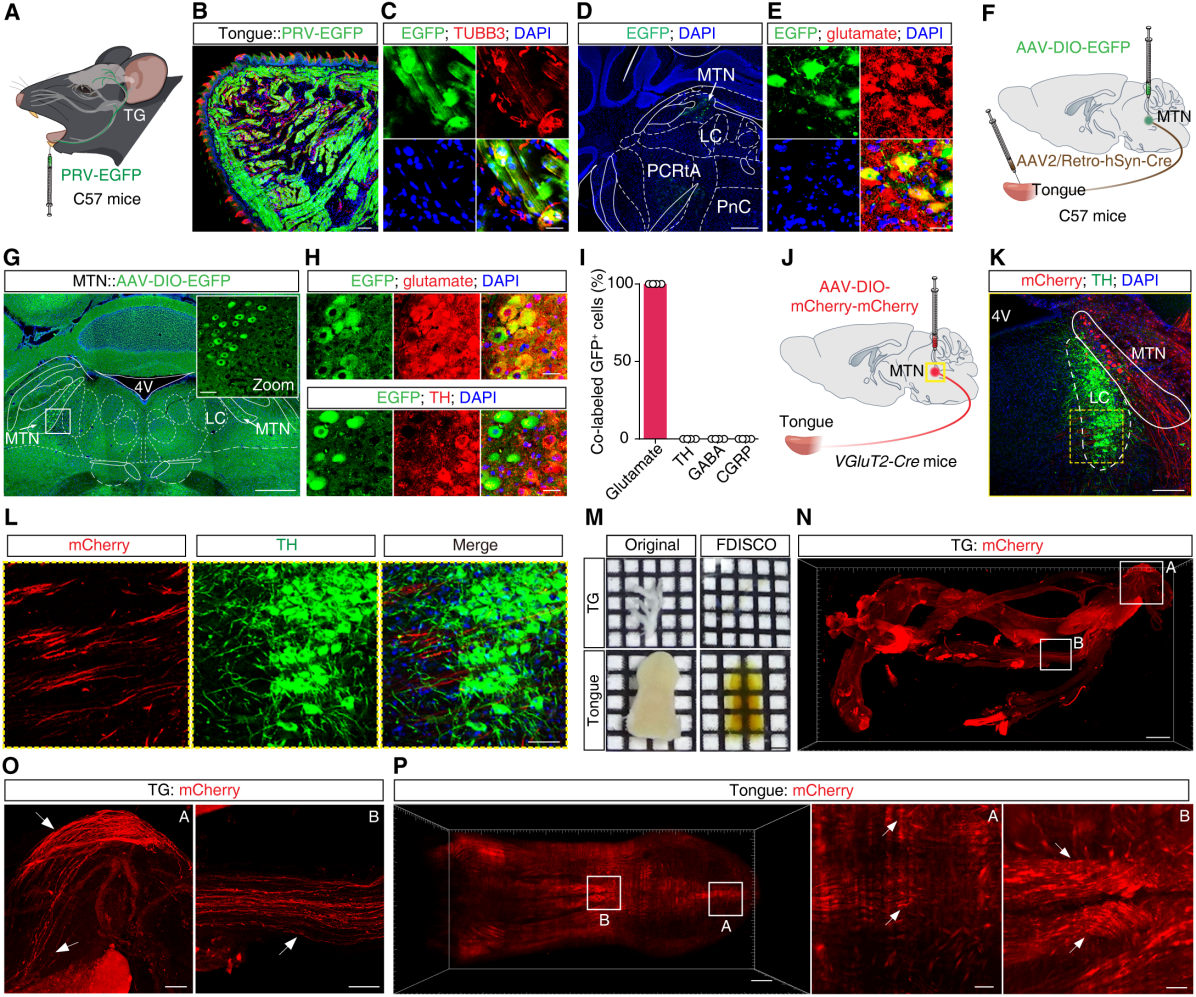
MTN^Glu^ neurons directly project to the tongue. (**A**) Schematic for retrograde transsynaptic tracing from the tongue to the MTN using PRV-EGFP. (**B** and **C**) Representative images of the tongue injection site of PRV-EGFP (B). The inset depicts the area shown in the white box, in which EGFP^+^ cells colocalized with an antibody against TUBB3 (C). Scale bars, 100 μm (overview) and 20 μm (inset). (**D** and **E**) Representative images showing EGFP^+^ neurons in the MTN (D); these signals were colocalized with a glutamate-specific antibody (E). Scale bars, 500 μm (overview) and 20 μm (inset). (**F**) Schematic for anterograde monosynaptic tracing from the tongue to the MTN. (**G**) Representative images showing viral expression in the MTN. Scale bars, 500 μm (overview) and 50 μm (inset). (**H** and **I**) Representative images (H) and quantification analysis (I) showing the EGFP-labeled tongue-projecting MTN neurons colocalized with a glutamate-specific antibody but not with a TH antibody. Scale bars, 20 μm. *n* = 4 mice. (**J**) Schematic diagram of AAV-DIO-mCherry-mCherry virus injection in the MTN of *VGluT2-Cre* mice. (**K** and **L**) Representative images of the injection site and viral expression within the MTN of *VGluT2-Cre* mice. Scale bars, 200 μm (overview) and 50 μm (zoom). (**M**) Representative images of intact, unsectioned tissue of trigeminal ganglion (TG) and tongue tissue before (original) and after (FDISCO) inducing translucency. Scale bar, 2.3 mm. (**N** to **P**) Representative images of whole TG (N and O) and tongue (P) sections showing the mCherry signal with AAV-DIO-mCherry-mCherry virus injection in the MTN. Scale bars, 500 μm. The zoom images are magnified views from the white-boxed regions in the TG and tongue. Scale bars, 100 μm. Data are presented as the means ± SEMs.

We also used a virus-based approach to examine neural connections between the MTN and the tongue, specifically by infusing a retrograde trans-synaptic pseudorabies virus [PRV–enhanced green fluorescent protein (EGFP)] into the tongue. One week later, EGFP^+^ signals were observed in multiple nuclei, including the MTN (Fig. 2, A to C, and fig. S6). Further immunofluorescence staining showed colabeled EGFP^+^ neurons positive for a glutamate-specific antibody (Fig. 2, D and E). To specifically label and characterize the tongue projecting somata in the MTN, we injected an AAV2/Retro-hSyn-Cre virus into the tongue and an AAV-DIO-EGFP virus into the MTN of C57 mice (Fig. 2F). Three weeks later, abundant EGFP^+^ neurons were observed in the MTN, which were colocalized specifically with an antibody marking glutamatergic neurons, but not antibodies for γ-aminobutyric acid (GABA)–, TH-, or calcitonin gene–related peptide-specific antibodies (Fig. 2, G to I, and fig. S7), suggesting that MTN^Glu^ neurons directly project to the tongue. Moreover, we bilaterally infused an anterograde AAV-DIO-mCherry-mCherry virus into the MTN of *VGluT2-Cre* mice (Fig. 2, J to L). After 1 month of viral expression, we used a solvent-based clearing method for imaging whole organs (FDISCO, DISCO with superior fluorescence-preserving capability) (*32*), which rendered the tissue largely translucent, to facilitate whole-tissue imaging of the intact, unsectioned trigeminal ganglion and tongue (Fig. 2M). We observed abundant mCherry^+^ MTN^Glu^ neuronal terminals in the mandibular branch (V3) of the trigeminal ganglion and the tongue (Fig. 2, N to P; and movies S3 and S4). Notably, we also detected abundant mCherry^+^ nerve fibers in the LC adjacent to the MTN (Fig. 2L). Together, these findings establish the presence of a MTN^Glu^ → tongue circuit.

### Rescue-like behavior increases LC^NE^ neuronal activity in anesthetized mice

**Fig. 3.**
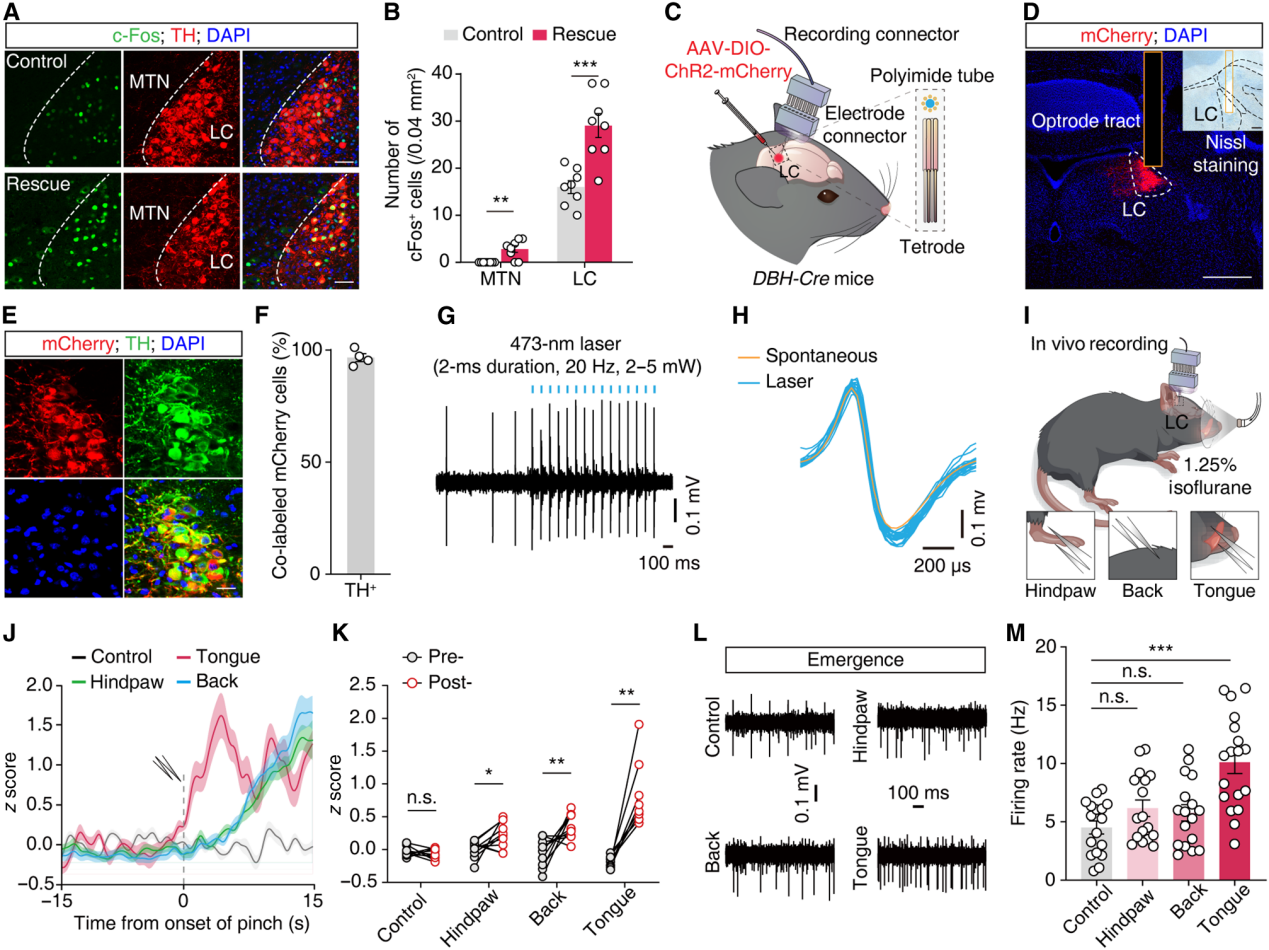
The rescue-like behavior increases LC^NE^ neuronal activity. (**A** and **B**) Representative images (A) and quantification (B) of c-Fos^+^ neurons in the MTN and the LC of AM in the presence (rescue) or absence (control) of bystander mice. Scale bars, 50 μm. *n* = 8 slices from four mice. (**C**) Schematic diagram of in vivo electrophysiological recording with an optrode. (**D**) Representative images of the virus injection site and Nissl staining of the optrode tract in the LC. Scale bars, 500 μm (overview) and 100 μm (zoom). (**E** and **F**) Representative images (E) and quantitative data (F) showing that mCherry^+^ neurons were colocalized with a TH-specific antibody in the LC. Scale bar, 20 μm. (**G** and **H**) Example recording of spontaneous and light-evoked spikes from LC^NE^ neurons (G) and overlay of averaged spontaneous (yellow) and light-evoked (blue) spike waveforms from the example unit (H). (**I**) Schematic for in vivo electrophysiological recording in isoflurane-AM with or without (control) the application of pinch stimuli to the tongue, hindpaw, and back skin during the emergence period, respectively. (**J** and **K**) Representative traces (J) and responses (K) of LC^NE^ neurons from control, pinched hindpaw, back, and tongue of AM during the emergence period. The dashed line indicates the onset of pinching stimuli. The bold line and light shadow indicate the mean and SEM, respectively. *n* (control, hindpaw, back, and tongue) = 9, 10, 10, and 8 units from 4 mice, respectively. (**L** and **M**) Raster plots with typical traces (L) and quantitative data (M) of spontaneous firing rates of LC^NE^ neurons in AM. *n* (control, hindpaw, back, and tongue) = 17, 17, 17, and 18 units from four mice, respectively. Data are presented as the mean ± SEMs. *P < 0.05, **P < 0.01, and ***P < 0.001. Details of the statistical analyses are presented in table S1. DAPI, 4′,6-diamidino-2-phenylindole.

Previous studies have reported that MTN neurons send projections to the LC, a brainstem region known to modulate arousal (*33*–*35*). The LC has extensive connectivity with various brain regions through its noradrenergic output, which enables it to respond to a wide range of sensory stimuli and influence the autonomic nervous system (*36*, *37*). We then examined how MTN^Glu^ and LC^NE^ neuronal activity changed in anesthetized mice after being rescued by bystanders. First, we investigated the neuronal activity of MTN and LC in anesthetized mice through observation of c-Fos expression, finding that the MTN and LC of anesthetized mice with bystanders had a significantly larger number of c-Fos^+^ neurons than that in the controls without bystanders (Fig. 3, A and B). These results suggest that the rescue-like behavior activated MTN^Glu^ and LC^NE^ neurons in anesthetized mice.

Seeking to replicate the neuronal activity changes observed in anesthetized mice triggered by the rescue-like behavior (tongue dragging) from bystanders, we used in vivo multi-tetrode recordings with an optrode to measure LC^NE^ neuronal activity during administration of a pinch stimulus to the tongue in anesthetized mice (Fig. 3C). The characteristics of spike waveforms of LC^NE^ neurons were identified by using optogenetic tagging in *DBH-Cre* mice, a transgenic mouse line with Cre recombinase expressed under the control of the dopamine-β-hydroxylase (DBH) promoter (Fig. 3, D to H) (*38*). We found that the spontaneous firing rates of LC^NE^ neurons were significantly increased following hindpaw, back skin, and tongue pinching of anesthetized mice (Fig. 3, I to K); however, this increase was lower in magnitude and exhibited a significant delay compared to the response elicited by tongue pinching (Fig. 3, J to M, and fig. S8, A to D), while no notable difference was observed between the control group and other groups in the induction and maintenance period (fig. S8, E to H). These results suggest that tongue pinching apparently has a greater arousal-promoting effect than pinching of other body parts.

### A tongue → MTN^Glu^ → LC^NE^ circuit controls rescue-like behavior-induced arousal

**Fig. 4.**
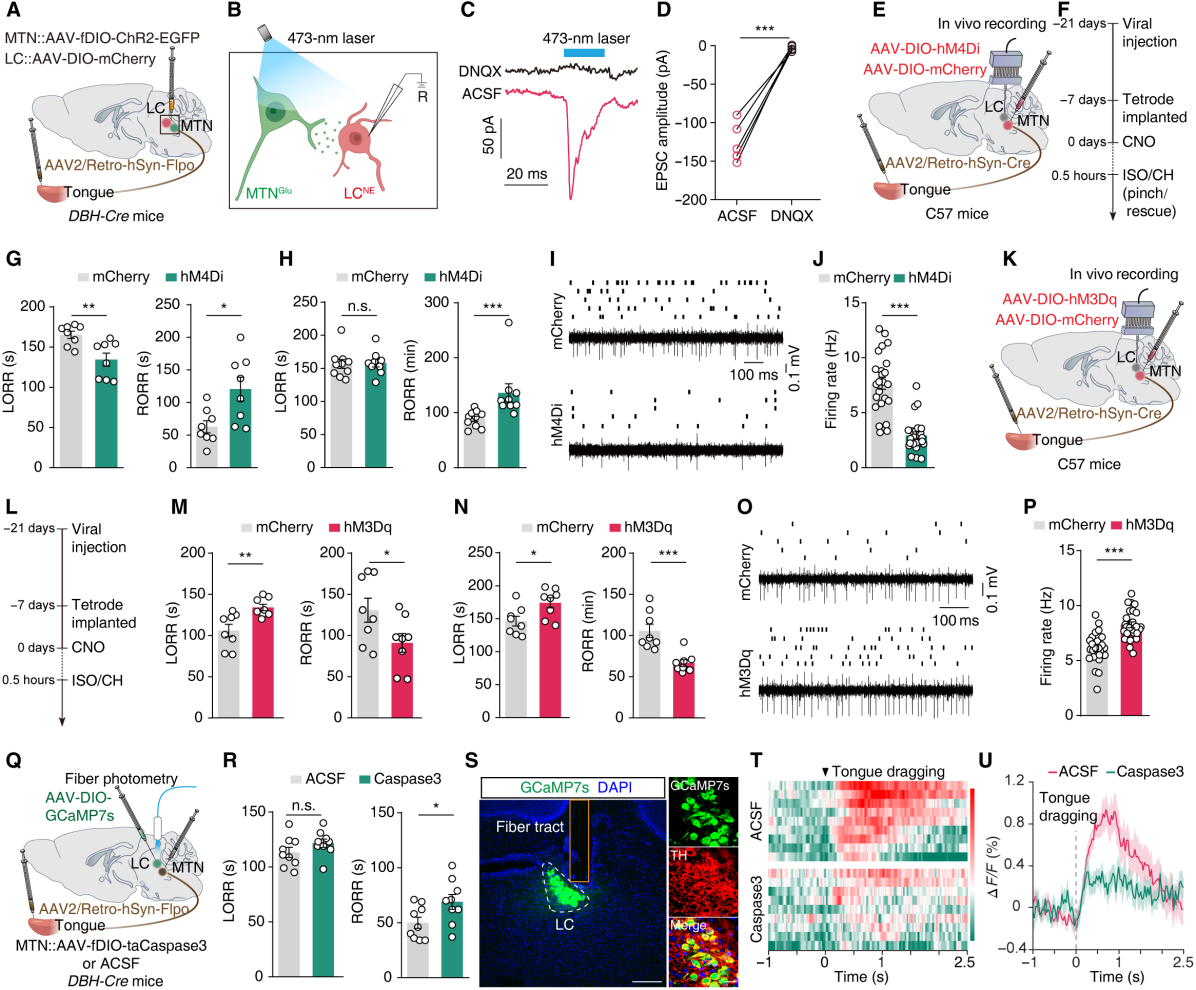
The tongue-projecting MTN^Glu^ controls arousal. (**A** and **B**) Schematic for optogenetics and whole-cell patch-clamp recordings. (**C** and **D**) Representative traces and summary data for light-evoked postsynaptic currents recorded from LC^NE^ neurons. *n* = 5. (**E** and **F**) Schematic and timeline of chemogenetic inhibition and recording in vivo. (**G**) Summary data for LORR and RORR from isoflurane-anesthetized mCherry and hM4Di mice after tongue pinching during the emergence period. *n* = 8. (**H**) Summary data for LORR and RORR from chloral hydrate-anesthetized mCherry and hM4Di mice after received rescue-like behavior from bystander mice. *n* (mCherry) = 10, *n* (hM4Di) = 9. (**I** and **J**) Raster plots with typical traces and quantitative data of spontaneous firing rates of LC^NE^ neurons from indicated group. *n* (mCherry and hM4Di) = 24 and 23 units from four mice, respectively. (**K** and **L**) Schematic and timeline of chemogenetic activation and recording in vivo. (**M** to **P**) As indicated in (G) to (J), but for mCherry and hM3Dq mice. *n* = 8 for (M) and (N), while *n* (mCherry and hM3Dq) = 24 and 25 units from 4 mice for (P), respectively. (**Q**) Schematic of virus injection and fiber photometry recordings in vivo in *DBH-Cre* mice. (**R**) Summary data for LORR and RORR from isoflurane-anesthetized ACSF and taCaspase3 mice after tongue pinching during the emergence period. *n* = 9. (**S**) Representative images validate GCaMP7s expression in TH^+^ neurons. Scale bars, 200 μm (overview) and 20 μm (inset). (**T**) Heatmaps across animals aligned to the time from onset of bystander mice engage in tongue dragging toward AM. (**U**) Average Δ*F*/*F* of LC-NE^GCaMP7s^ signals from indicated group. Data are presented as the means ± SEMs. *P < 0.05, **P < 0.01, and ***P < 0.001. Details of the statistical analyses are presented in table S1.

Previous studies have demonstrated that the presence of pseudounipolar neurons within the MTN (*39*, *40*). To investigate the functional connections between the MTN and LC, we infused an AAV2/Retro-hSyn-Flpo virus into the tongue and a mixture of AAV-DIO-mCherry and AAV-fDIO-ChR2-GFP viruses into the MTN (adjacent to the LC) of *DBH-Cre* mice (Fig. 4, A and B). Under whole-cell voltage clamp at −70 mV, photostimulation of ChR2-expressing MTN^Glu^ terminals in the LC evoked reliable excitatory postsynaptic currents (EPSCs) in LC^NE^ neurons (Fig. 4C). These EPSCs could be blocked by the AMPA receptor antagonist 6,7-dinitroquinoxaline-2,3-dione (DNQX) (Fig. 4D). These findings demonstrate that LC^NE^ neurons receive direct monosynaptic input from tongue-projecting MTN^Glu^ neurons.

To assess the potential role of MTN^Glu^ neurons in modulating arousal in anesthetized mice, we used designer receptors exclusively activated by designer drugs (DREADDs) to selectively inhibit or activate these neurons. Specifically, we infused a retrograde AAV2/Retro-hSyn-Cre virus into the tongue and a Cre-dependent AAV-DIO-hM4Di-mCherry virus into the bilateral MTN of C57 mice (Fig. 4, E and F). Three weeks later, whole-cell patch-clamp recordings in brain slices showed that the hM4Di-expressing neurons in the MTN were functionally hyperpolarized following incubation with clozapine-N-oxide (CNO, 10 μM) (fig. S9, A to C). Chemogenetic inhibition of the tongue-projecting MTN neurons significantly decreased the LORR while significantly increased the RORR in hM4Di-expressing anesthetized mice as compared to mCherry-expressing controls (Fig. 4, G and H). In vivo multi-tetrode recordings showed that the spontaneous firing rates of LC^NE^ neurons were significantly decreased in hM4Di-expressing mice compared with mCherry-expressing controls upon pinching tongue during the emergence period (Fig. 4, I and J). By contrast, chemogenetic activation of the tongue-projecting MTN neurons led to a significant increase in the LORR and a significant decrease in the RORR in anesthetized mice (Fig. 4, K to N, and fig. S9, D to F). This activation also resulted in significantly elevated firing rates of LC^NE^ neurons in hM3Dq-expressing mice compared with mCherry-expressing controls (Fig. 4, O and P). These results demonstrate that the MTN^Glu^ neurons are required for the rapid arousal effect induced by rescue-like behavior.

Next, we directly assessed whether the MTN^Glu^ neurons contribute to the facilitated arousal effect of rescue-like behavior by modulating LC^NE^ neuronal activity, specifically by ablating MTN^Glu^ neurons based on the infusion of a AAV2/Retro-hSyn-Flpo virus into the tongue and a Flpo-dependent genetically engineered caspase3 (AAV-fDIO-taCaspase3) into the MTN of C57 mice (Fig. 4Q and fig. S9G). The activation of taCaspase3 has been shown to induce cell apoptosis (*41*). Four weeks later, immunofluorescence staining showed that MTN^Glu^ neurons were selectively ablated, while the glutamatergic neurons of the LC remained intact (fig. S9, H and I). Mice lacking MTN^Glu^ neurons exhibited a significant increase in the RORR for isoflurane anesthesia, while no significant difference in the LORR was observed (Fig. 4R). Moreover, in vivo fiber photometry recordings showed that the calcium activity of LC^NE^ neurons was significantly decreased in taCaspase3-expressing anesthetized mice compared with artificial cerebrospinal fluid (ACSF)–treated control mice upon rescued by bystander mice (Fig. 4, S to U). Thus, the rescue-like behavior that promotes arousal in anesthetized mice increases activity of the tongue → MTN^Glu^ → LC^NE^ circuit.

### Glutamatergic neurons in the PVT are functionally connected to the NAcSh

After identifying the MTN^Glu^ neurons that innervate the tongue and monosynaptically connect with LC^NE^ neurons, thereby rapidly modulating arousal levels through tongue-dragging behavior from the bystanders, we turned our focus to the brain regions potentially contributing to the rescue-like behavior of the bystander mice. We used a screening approach based on the detection of c-Fos—an immediate-early marker of neuronal activity—to define relevant brain areas associated with the rescue-like behavior. We found that the PVT and NAcSh were significantly activated in bystander mice exhibiting rescue-like behavior (fig. S10). Subsequent immunofluorescence staining revealed that c-Fos colocalized with approximately 94% of glutamatergic neurons in PVT and 87% of GABAergic neurons in NAcSh (fig. S11).

As the PVT and NAc-related projections have been shown to be involved in motivated and consolatory behaviors (*42*–*44*), we speculated that the PVT → NAcSh circuit might also be involved during rescue-like behavior by the bystander mice. To investigate the precise cell type–specific organization of this connection, AAV2/Retro-hSyn-EGFP was injected into the NAcSh (fig. S12A). Abundant EGFP^+^ neurons were observed in the PVT, which were entirely colabeled with a glutamatergic-specific antibody (fig. S12, B and C). On the other hand, to characterize the composition of NAcSh neurons receiving PVT projections, we performed anterograde monosynaptic tracing by PVT injection with AAV2/1-hSyn-Cre-EGFP along with ipsilateral NAcSh injection of AAV-DIO-mCherry and found the abundant mCherry^+^ neurons, which were colocalized with GABAergric neurons in the NAcSh (fig. S13, A to C). Moreover, mCherry^+^ terminals originating from the NAcSh were observed in multiple other brain regions, including the anterior hypothalamic nucleus (fig. S13D), which has been previously implicated in biting attacks behavior (*28*).

**Fig. 5.**
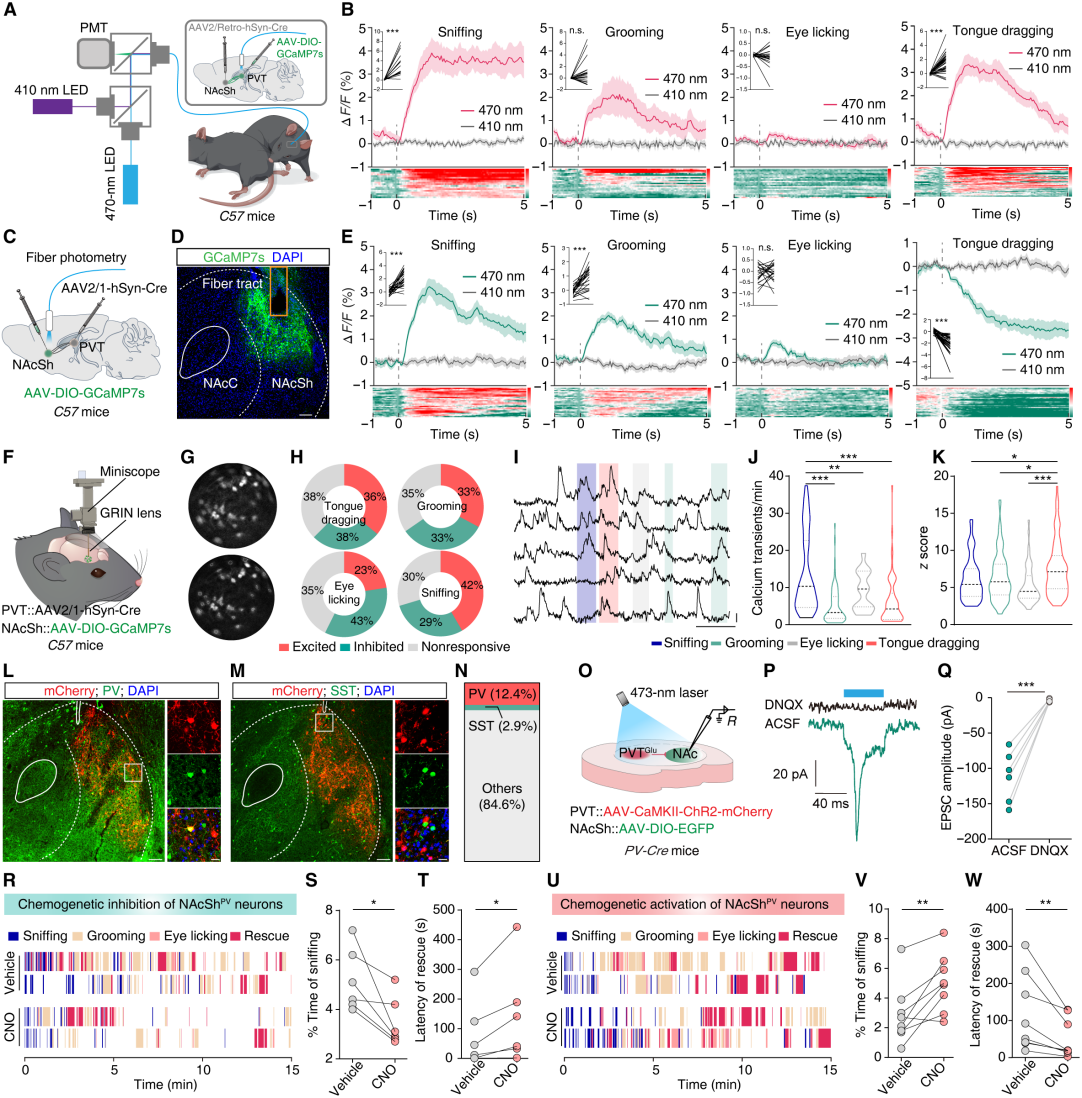
NAcSh^PV^ neurons regulate the latency of rescue-like behavior in bystander mice. (**A** and **B**) Schematic of fiber photometry and representative traces with summary Δ*F*/*F* signals from NAcSh-projecting PVT^Glu^ neurons when bystander mice engaged in various behavior. Heatmaps across trials aligned to the time from onset of various behavior (bottom). *n* (sniffing, grooming, eye licking, and tongue dragging) = 18, 21, 22, and 33 trails from five mice, respectively. (**C** to **E**) As indicated in panels A and B, but for PVT-innervated NAcSh neurons. *n* = 26, 26, 24, and 32 trails from five mice, respectively. (**F** and **G**) Schematic for microendoscopic imaging and automatically extracted single neurons from imaging view in the NAcSh. (**H**) Pie chart showing the proportion of NAcSh^GCaMP7s^ neurons responding to various behaviors. (**I** to **K**) Example spontaneous Δ*F*/*F* time-series traces and summary data for calcium transients and average Δ*F*/*F* responses of NAcSh^GCaMP7s^ neurons. The black and gray dashed lines indicate the mean and SEM, respectively. *n* (sniffing, grooming, eye licking, and tongue dragging) = 62, 84, 46, and 33 cells from three mice, respectively. (**L** to **N**) Typical images and summary data for the PVT-innervated NAcSh^mCherry^ neurons were stained with a PV- or somatostatin (SST)–specific antibody. Scale bars, 100 μm (overview) and 20 μm (inset). (**O**) Schematic for optogenetics and whole-cell patch-clamp recordings. (**P** and **Q**) Representative traces and summary data for light-evoked postsynaptic currents recorded from NAcSh^PV^ neurons. *n* = 6. (**R** to **W**) Example raster plots and summary data for hM4Di- or hM3Dq-expressing bystander mice engaged in various behavior after treatment with vehicle or CNO. *n* = 6 for panels S and T; *n* = 8 for (V) and (W). Data are presented as the means ± SEMs. *P < 0.05, **P < 0.01, and ***P < 0.001. Details of the statistical analyses are presented in table S1.

To investigate whether the neuronal activity of glutamatergic neurons in the PVT (PVT^Glu^) projecting to the NAcSh is altered when bystander mice engage in the rescue-like behavior, we performed fiber photometry recordings in freely moving C57 mice injected with AAV2/Retro-hSyn-Cre in the NAcSh along with ipsilateral PVT injection of AAV-DIO-GCaMP7s (Fig. 5A). The calcium activity of GCaMP7-expressing PVT neurons was significantly increased in bystander mice when they displayed sniffing, grooming, and tongue dragging to anesthetized mice, while no notable difference was observed in the eye licking behavior (Fig. 5B). These findings indicate the activation of NAcSh-projecting PVT^Glu^ neurons when bystander mice engage in the rescue-like behavior.

### Activation of the PVT^Glu^ → NAcSh circuit meditates rescue-like behavior

To investigate the function of PVT-NAcSh circuit in rescue-like behavior, we used a DREADD approach. To this end, we performed bilateral injection of an AAV-DIO-hM4Di-mCherry virus into the PVT and implanted guide cannulas to locally perfuse CNO into the NAcSh of Ca^2+^/calmodulin-dependent protein kinase II (CaMKII, an enzyme in glutamatergic neurons)–*Cre* mice (fig. S14A) (*45*). Chemogenetic inhibition of the PVT^Glu^ → NAcSh circuit resulted in a significant reduction of rescue-like behavior in bystander mice (fig. S14, B to G), while the chemogenetic activation of this circuit significantly increased the duration and number of occurrences of rescue-like behavior (fig. S14, H to N); no CNO-induced difference was observed in mCherry-expressing control mice (fig. S14, D to G and K to N). These findings suggest that the PVT → NAcSh circuit is required for the rescue-like behavior of bystander mice.

To dynamically visualize the calcium activity of PVT-innervated NAcSh neurons in freely moving mice, an anterograde AAV2/1-hSyn-Cre virus was injected into the PVT in combination with AAV-DIO-GCaMP7s virus that was injected ipsilaterally into the NAcSh of C57 mice (Fig. 5, C and D). Fiber photometry recordings showed that the calcium activity of GCaMP7-expressing NAcSh neurons was significantly increased when the bystander mice engaged in sniffing and grooming behaviors; no difference in calcium activity was evident during eye licking behavior (Fig. 5E). Calcium activity was significantly decreased in the bystander mice exhibiting the rescue-like behavior (Fig. 5E). In light of previous findings reporting functional inputs from the PVT to striatal PV-expressing INs (*46*), we speculated that the NAcSh^PV^ neurons may play a role in the rescue-like behavior.

Given that fiber photometry recordings capture only changes in the calcium activity of the NAcSh neuronal population, we conducted microendoscopic calcium imaging to selectively monitor the activity of PVT-innervated NAcSh neurons at single-neuron resolution, specifically as bystander mice engage in the rescue-like behavior. We infused AAV2/1-hSyn-Cre into the PVT and AAV-DIO-GCaMP7s into the ipsilateral NAcSh, accompanied with the mounting of a microendoscopic gradient index (GRIN) lens at the top of the NAcSh (Fig. 5, F and G). As bystander mice engaged in the rescue-like behavior, we detected three trends of PVT-innervated NAcSh neurons, which showed an increase (36%), a decrease (38%), or no change (38%) (Fig. 5H and movie S4), suggesting that a subpopulation of neurons within the NAcSh were activated by receiving directly excitatory projections from the PVT at the onset of rescue-like behavior. We also detected three trends in PVT-innervated NAcSh neurons during sniffing (excited: 42%, inhibited: 29%, and nonresponsive: 30%), grooming (excited: 33%, inhibited: 33%, and nonresponsive: 35%), and eye licking (excited: 23%, inhibited: 43%, and nonresponsive: 35%) (Fig. 5H). Moreover, we found that the calcium transients of excited neurons were significantly greater when bystander mice displayed sniffing than the other three behaviors, while the calcium activity of excited neurons was significantly higher when bystander mice engaged in rescue-like behavior than in the other three behaviors (Fig. 5, I to K). These results are consistent with the fiber photometry recording data, indicating that the highest NAcSh neuronal activity occurred during sniffing. Although fewer NAcSh neurons were activated during tongue-dragging behavior, the calcium activity in these neurons was higher than during the other behaviors in bystander mice, implying that a small proportion of PVT-innervated NAcSh neurons may specifically respond to rescue-like behavior.

Next, to explore the specific types of neurons in the NAcSh involved in rescue-like behavior, we performed anterograde monosynaptic tracing of the PVT → NAcSh circuit (fig. S13B). Subsequent immunofluorescence staining showed that 12.4% of mCherry^+^ neurons were costained with a PV-specific antibody, only 2.9% of mCherry^+^ neurons colocalized with a somatostatin-specific antibody (Fig. 5, L to N). Then, to investigate the functional connections of the PVT^Glu^ → NAcSh^PV^ circuit, we infused AAV-CaMKII-ChR2-mCherry virus into the PVT and simultaneously injected AAV-DIO-EGFP virus into the NAcSh of *PV-Cre* mice to label NAcSh^PV^ neurons (Fig. 5O). Under whole-cell voltage clamp at −70 mV, photostimulation of ChR2-containing PVT^Glu^ terminals in the NAcSh evoked reliable EPSCs from the EGFP^+^ NAcSh^PV^ neurons (Fig. 5P). These EPSCs could be blocked by the AMPA receptor antagonist DNQX (Fig. 5Q), indicating that PVT^Glu^ neurons send excitatory projections to regulate NAcSh^PV^ neuronal activity.

To examine whether NAcSh^PV^ neurons modulate rescue-like behaviors of bystander mice, we infused a Cre-dependent AAV-DIO-hM4Di-mCherry virus into the bilateral NAcSh of *PV-Cre* mice. Three weeks later, chemogenetic inhibition of NAcSh^PV^ neurons significantly decreased the duration and frequency of sniffing yet significantly increased the latency of the tongue-dragging behavior in bystander mice (Fig. 5, R to T, and fig. S15, A and B). Conversely, the chemogenetic activation of NAcSh^PV^ neurons resulted in a significant increase in both the duration and frequency of sniffing and a significant decrease in the latency of the tongue-dragging behavior in bystander mice (Fig. 5, U to W, and fig. S15, C and D). Notably, the chemogenetic modulation of NAcSh^PV^ neuronal activity does not affect the locomotor ability of the mice (fig. S15, E to G). These results thus demonstrate that the PVT^Glu^ → NAcSh^PV^ circuit controls the rescue-like behavior in bystanders.

### Activation of the NAcSh^D1-MSN^ neurons increase rescue-like behavior

**Fig. 6.**
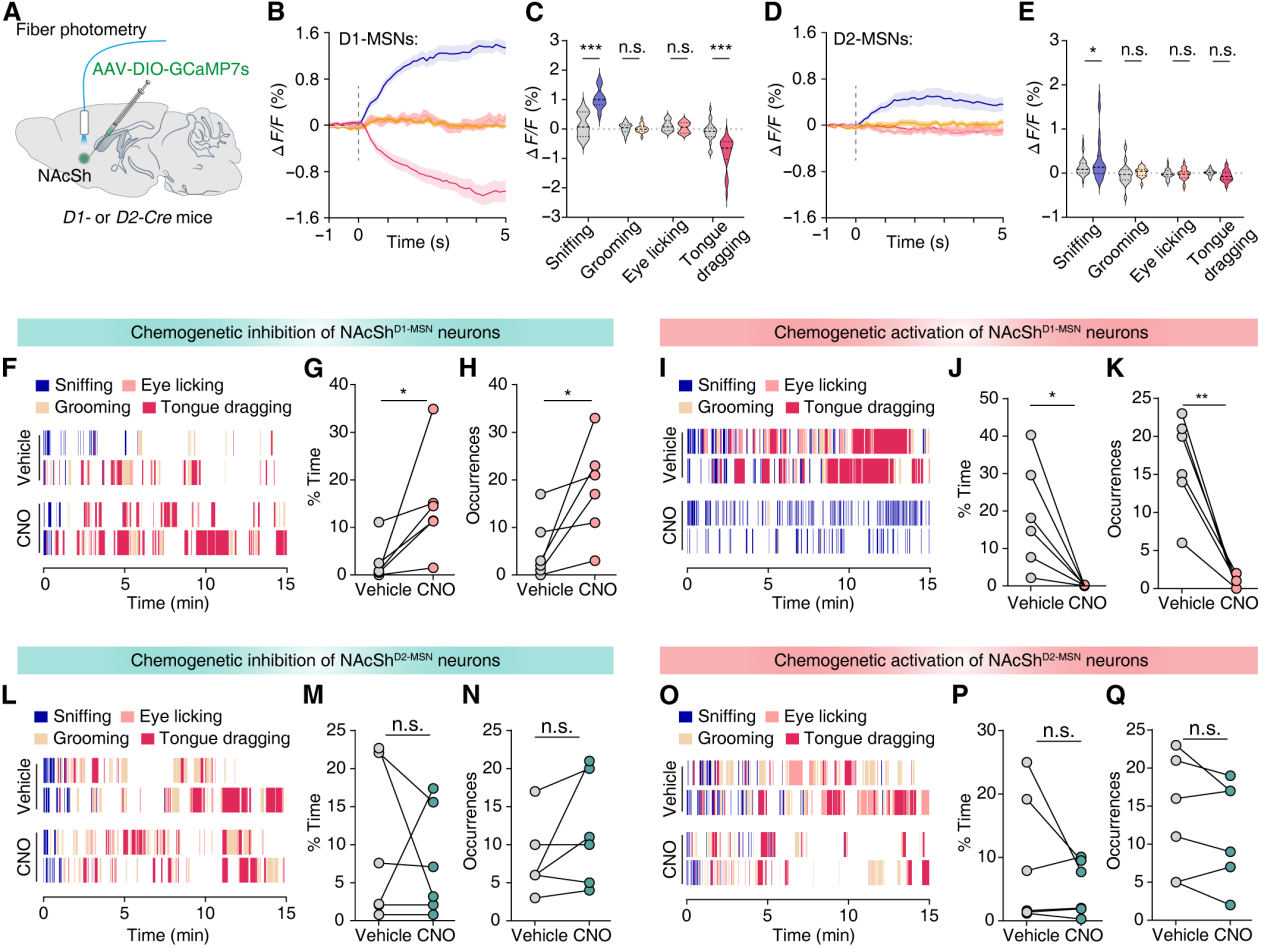
NAcSh^D1-MSN^ but not NAcSh^D2-MSN^ neurons regulate tongue-dragging behaviors in bystander mice. (**A**) Schematic of fiber photometry recording. (**B** and **C**) Representative traces and average Δ*F*/*F* signals of NAcSh^D1-MSN^ neurons when bystander mice engaged in various behavior. The bold line and light shadow indicate the means and SEM, respectively. *n* (sniffing, grooming, eye licking, and tongue dragging) = 18, 18, 8, and 23 trails from five mice, respectively. (**D** and **E**) As indicated in (B) and (C), but for NAcSh^D2-MSN^ neurons. *n* (sniffing, grooming, eye licking, and tongue dragging) = 32, 18, 23, and 32 trails from five mice, respectively. (**F**) Example raster plots showing bystander mice engaged in various behaviors after chemogenetic inhibition of NAcSh^D1-MSN^ neurons. (**G** and **H**) Total duration percentage (G) and the number of occurrences (H) of tongue-dragging behavior in hM4Di-expressing bystander mice after treatment with vehicle or CNO. *n* = 6 mice. (**I** to **K**) As indicated in (F) to (H), example raster plots and summary data from bystander mice following chemogenetic activation of NAcSh^D1-MSN^ neurons. *n* = 6 mice. (**L** to **Q**) As indicated in (F) to (K), but for chemogenetic inhibition or activation of NAcSh^D2-MSN^ neurons. *n* = 6 mice. Data are presented as the means ± SEMs. *P < 0.05, **P < 0.01, and ***P < 0.001. Details of the statistical analyses are presented in table S1.

In the NAc, D1- and D2-MSNs were synaptically innervated by PVT^Glu^ neurons (*46*). Therefore, we aimed to determine whether these two populations of neurons in the NAcSh modulate the rescue-like behavior in bystander mice. We injected AAV-DIO-GCaMP7s virus into the NAcSh of *D1-Cre* and *D2-Cre* mice and used fiber photometry to record changes in calcium signals from the two neuronal populations *in vivo* (Fig. 6A). Fiber photometry recordings showed a significant decrease in calcium activity of D1-MSNs when bystander mice engaged in tongue-dragging behaviors toward anesthetized mice, while a significant increase was observed when bystanders displayed sniffing (Fig. 6, B and C). Notably, we only detected a significant increase in calcium activity of D2-MSNs when bystander mice exhibited sniffing, while no changes in other three behaviors (Fig. 6, D and E). These findings indicate that the NAcSh^D1-MSN^ neuronal activity is inhibited when bystander mice engage in rescue-like behavior.

To further investigate the role of NAcSh^D1-MSN^ and NAcSh^D2-MSN^ neurons in the rescue-like behaviors of bystander mice, we bilaterally infused AAV-DIO-hM4Di-mCherry or AAV-DIO-hM3Dq-mCherry viruses into the NAcSh of *D1-Cre* and *D2-Cre* mice (Fig. 6, F to Q). Behavioral results showed that inhibiting D1-MSNs significantly increased rescue-like behaviors in bystander mice, with increases evident in both the duration and the frequency of tongue dragging (Fig. 6, F to H). Conversely, chemogenetic activation of D1-MSNs resulted in significant decreases in both the frequency and duration of tongue-dragging behavior in bystander mice (Fig. 6, I to K). Notably, both chemogenetic inhibition and activation of D2-MSNs had no notable effect on rescue-like behaviors in bystander mice (Fig. 6, L to Q). Together, these results suggest that NAcSh^D1-MSN^ but not NAcSh^D2-MSN^ neurons regulate rescue-like behaviors in bystander mice.

## DISCUSSION

**Fig. 7.**
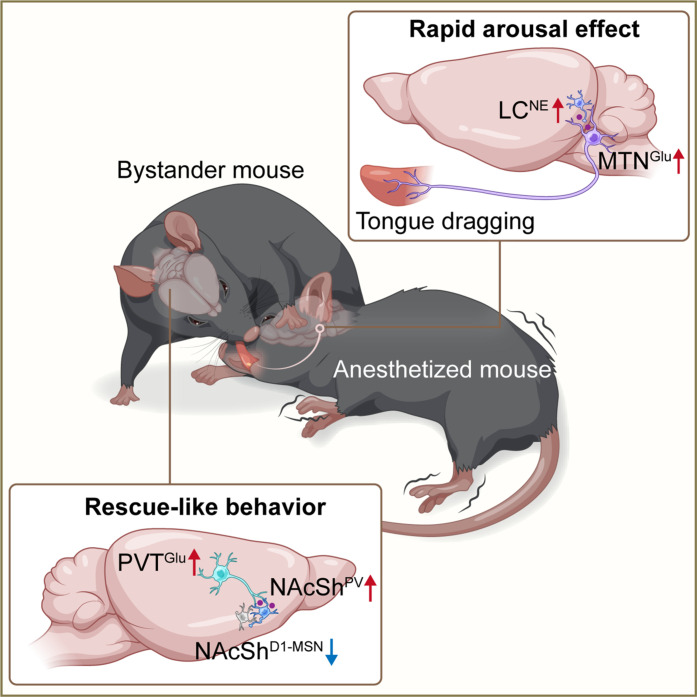
Rescue-like behavior promotes arousal via a tongue-brain circuit. The bystander mice engage in a form of rescue-like behavior (tongue dragging) toward other anesthetized mice, which aids the recipient’s arousal from general anesthesia. We found that a PVT^Glu^ → NAcSh^PV → D1-MSN^ circuit initiates the rescue-like behavior of bystander mice, while a direct neural circuit of tongue → MTN^Glu^ → LC^NE^ mediates the rapid arousal effect induced by the rescue-like behavior in anesthetized mice. PVT, paraventricular nucleus of the thalamus; NAcSh, nucleus accumbens shell; MTN, mesencephalic trigeminal nucleus; LC, locus coeruleus; Glu, glutamate; PV, parvalbumin; NE, norepinephrine; D1-MSN, dopamine-1 receptor–expressing medium spiny neuron.

In this study, we found that a PVT^Glu^ → NAcSh^PV → D1-MSN^ circuit drives rescue-like behavior in bystander mice. We also found that this rescue-like behavior facilitates the arousal of anesthetized cagemates through a direct tongue → MTN^Glu^ → LC^NE^ circuit (Fig. 7).

Prosocial behaviors that benefit others are essential for species survival. Mice have been shown to exhibit social transmission of pain and emotion in previous studies (*4*, *47*, *48*). In the present study, the bystander mice displayed specific tongue dragging toward the anesthetized mice—an observation that, to our knowledge, has not been reported previously. We found that this tongue dragging promotes arousal of anesthetized mice in a laboratory setting. Although its occurrence in wild conditions remains unknown, it may represent a form of prosocial behavior aimed at aiding an incapacitated companion. Recent studies have shown that rodents display several types of prosocial behaviors toward distressed partners. For example, motivated helping behavior was observed when cagemates were restrained, and bystander rats would open the restrainer to release their partner (*8*); it has also been reported that bystander mice exhibit affiliative allogrooming and social licking behavior toward distressed cagemates, resulting in alleviation of anxiety-associated behaviors and an increased ability to cope with pain in stressed partners (*5*, *11*). Notably, and unlike previous studies that required extensive training to elicit helping behaviors (*8*, *12*, *13*), the tongue-dragging behavior reported here emerged spontaneously in the laboratory. This paradigm provides a relatively rapid and easy-to-implement assay for investigating a rescue-like prosocial behavior.

Previous studies have reported a similar pattern of the familiarity effect in both fear observation and socially transferred pain paradigms. For the fear observation, an observer mouse’s response was measured while it witnesses another mouse experiencing a negative stimulus, typically a footshock. These studies have reported that familiarity plays a role, particularly in males, who exhibit a reduced response when tested with an unfamiliar mouse. In contrast, female mice did not display this reduction (*49*–*51*). However, in paradigms measuring emotional contagion through pain hypersensitivity, female mice showed greater susceptibility to socially transferred pain hypersensitivity than males (*52*). This effect of sex-specific familiarity in social approach seems to be conserved across rodents, as a similar phenomenon has been observed in rats (*53*). Consistent with these evidences, our findings reveal a sex-specific familiarity effect, wherein females but not males are less likely to engage in this rescue-like behavior toward strangers.

Peripheral sensory stimuli are known to promote arousal from anesthesia (*37*, *54*). The tongue contains a rich distribution of sensory nerve fibers, and it has been shown that the MTN is an evolutionarily conserved nucleus that contains primary afferent sensory neurons, which are responsible for regulating the processing of sensory information from the tongue and the mouth (*55*–*57*). We found that a direct neural circuit from the tongue to the MTN is required for the rescue-like behavior to promote arousal in anesthetized mice by increasing LC^NE^ neuronal activity. This is reminiscent of previous findings that acupuncture at the Renzhong acupoint on the face could exert an arousing effect in traditional Chinese emergency medicine (*19*, *58*). Considering the extensive innervation of the face and tongue by the trigeminal nerve in both humans and rodents, this arousal pathway may be evolutionarily conserved.

The PVT participates in the maintenance of body homeostasis by integrating the information of internal state and external environment to guide adaptive behavioral responses (*44*). We found that a PVT → NAcSh circuit drives the initiation of a prosocial behavior and found that a subset of PVT-innervated NAcSh neurons responded differentially to the rescue-like behavior compared to three other examined behaviors (sniffing, grooming, and eye licking). This is consistent with previous findings that the PVT or NAc neurons of bystanders are activated upon visualizing a distressed partner or hearing a prosocial ultrasonic vocalization (*21*, *43*). Previous studies have shown that the expression of oxytocin receptors in the suprachiasmatic nucleus (SCN) is regulated by *clock* genes, which mediate individual phenotypic differences in prosociality (*59*). In addition, the SCN projects to the medial amygdala, a brain region associated with social behavior in mice (*60*). The anterior hypothalamic nucleus (AHN) has been implicated in fight-or-flight responses to predators and in the regulation of parental behavior (*28*, *61*, *62*). A recent study found that AHN spiking activity is generally reduced during social encounters (*63*). In line with these findings, we observed a significant increase in SCN activity and a significant decrease in AHN activity in bystander mice following their engagement in rescue-like behaviors, suggesting that both the SCN and AHN may be involved in modulating rescue-like behaviors.

Previous studies have shown that feedforward inhibition in the NAc occurs when glutamatergic afferents onto MSNs collateralize onto fast-spiking PV-INs, which exert GABAergic control over D1-MSN action potential generation (*64*). Another study examined the PVT → NAc-dependent suppression of sucrose seeking and reported an elevated glutamatergic excitatory synaptic drive at PVT → NAc^PV-IN^ synapses as compared with PVT → NAc^D1-MSN^ and PVT → NAc^D2-MSN^ synapses (*46*). We found that both NAcSh^PV^ INs and NAcSh^D1-MSN^ are involved in modulating rescue-like behaviors in bystander mice and that PV-INs may exert a local inhibitory effect on D1-MSNs. Moreover, our in vivo microendoscopic calcium imaging data showed that approximately 36% of NAcSh neurons were excited when bystander mice were engaged in rescue-like behaviors, while around 38% neurons were inhibited (Fig. 5H). It must be noted that fiber photometry recordings reflect changes in the calcium activity of the overall neuronal population within the NAcSh and do not capture activity at the level of individual neurons. Combining the previously reported findings with our current results, it seems that even if the PVT innervates only 12% of NAcSh^PV-IN^ neurons, then this may induce sufficiently strong feedback inhibition to reduce overall NAcSh activity. Given that hypothalamic oxytocin signaling is involved in prosocial behavior and that the PVT and NAc richly express oxytocin receptors (*7*, *65*–*67*), it will be interesting to explore potential connections between oxytocin and the rescue-like behavior.

It remains unknown whether similar rescue-like behaviors occur in mice under wild conditions. However, it is notable that both humans and non-human primates are known to exhibit prosocial behaviors to actively rescue anesthetized or unconscious companions. For example, humans may slap the face or shake the body to induce awakening (*68*), and primates frequently touch an anesthetized or comatose companion, lick or clean an unconscious companion’s body, and may even make grunting noises in an attempt to communicate (*15*, *69*). These observations suggest that the rescue-like behavior observed in mice when their companion is unconscious or anesthetized may be evolutionarily conserved across species. Whether the tongue → MTN^Glu^ → LC^NE^ circuit involved in the observed rescue-like behavior are present (and whether they mediate the same functions) in primates and/or humans to be determined.

## MATERIALS AND METHODS

### Animals

In the present study, C57BL/6J, *DBH-Cre*, *VGluT2-Cre*, *CaMKII-Cre*, *D1-Cre*, and *D2-Cre* mouse lines at the age of 8 to 12 weeks old were used for experiments. Mice were group-housed (4 to 5 per cage) in a stable environment (23° to 25°C ambient temperature and 50% humidity) unless implanted with cannulas or tetrode arrays. The mice were maintained under a 12-hour light/12-hour dark cycle (lights on from 7:00 to 19:00) with ad libitum access to water and food. To generate litters, two females were mated with one male in our facility. The male was removed 10 days later, and the females were kept separately 1 to 4 days before delivery. All mouse feeding and experiments were conducted in accordance with the requirements of the Animal Care and Use Committee of the University of Science and Technology of China (USTCACUC25080124017). Except for those exploring the effect of gender on rescue-like behaviors, male mice were selected throughout the study.

### Behavioral paradigms

All the behavioral tests were performed during the light phase (7:00 to 19:00). Operators were blinded to the experimental group when scoring.

#### Open field test

For the open field test (OFT), an open field apparatus chamber (50 cm by 50 cm by 40 cm) consisting of a square central area (25 cm by 25 cm) and a marginal area (50 cm by 50 cm) was used. The mice were quickly placed in the central area of the test box and allowed to move freely, and a toy mouse or a chloral hydrate-anesthetized mouse was placed in the center and then recorded for 5 min. The inner wall and bottom of the test chamber were cleaned with 75% ethanol between tests to eliminate odor cues. The number of mouse entries in the central area, the total time spent in the central area, and the total distance traveled were analyzed using EthoVision XT 14 animal behavior analysis software.

#### Anesthesia behavior test

Mice were handled and habituated to the test arena for at least 2 days before the experiments and then moved to the behavioral room for at least 30 min before the experiments were performed. Isoflurane and chloral hydrate were used for short-term and long-term anesthesia tests, respectively. For isoflurane, 2.5 and 1.25 vol % isoflurane (RWD Life Science Inc., China, 0.7 liter/min, 15 min) were used in the short-term anesthesia test, and the latencies to LORR and RORR were recorded to evaluate the induction time and emergence time of the mice. For chloral hydrate, fresh chloral hydrate (Macklin) was dissolved in saline [5% (w/v) in 0.9% NaCl] and intraperitoneally injected at 400 mg/kg of body weight during long-term anesthesia to record the latencies of the LORR and RORR.

#### Rescue-like behavior test

For the rescue-like behavior test, the bystander mice were first placed in the experimental box (25 cm by 25 cm by 25 cm, made of transparent acrylic sheet), and then one chloral hydrate-anesthetized mouse was gently placed in the center of the box. Then, we started to record the rescue-like behavior of the bystander mice for 15 min. All the bystander mice were put into the box for at least 3 hours at night with a layer of new sawdust bedding, and the mice were put into the same box the next day for at least 1 hour before testing. The experiment was recorded by a video camera (X2S 4K, ONTOP) mounted above the experimental apparatus, and we can clearly observe that the anesthetized mice exhibiting leg shaking and body trembling when the bystander mice perform tongue-dragging behavior (i.e., the bystander mouse uses its teeth to grasp the tongue of the anesthetized mouse until the tongue protrudes from the mouth, followed by the use of the teeth to repeat “drag” the tongue of the anesthetized mouse). On the basis of these behavioral responses, we quantified the duration and frequency of tongue-dragging behavior using BORIS software.

### EEG/EMG recording and analysis

To implant EEG-EMG electrodes, four holes were drilled with metal bit: two over the frontal cortical area [anterior/posterior (A/P) = +1.60 mm; medio-lateral (M/L) = ±1.65 mm] and two over the parietal area (A/P = −3.7 mm; M/L = ±1.65 mm). Four screws were implanted into the holes to fix a head-mounted preamplifier that simultaneously recorded EEG and EMG signals (Pinnacle Technologies Inc, USA) and served as recording electrodes. Dental cement was eventually used to cover the whole EEG-EMG electrodes system. After the surgery, mice were placed on an electric warm blanket until fully awake. The raw EEG/EMG data were converted in NeuroExplorer 5 and exported to MATLAB (MathWorks). Data analyses and plots were produced using GraphPad Prism 8.0 (GraphPad Software, San Diego, CA, USA). In brief, wakefulness is characterized by desynchronized small-amplitude EEG and extensive EMG activity. The anesthesia test with EEG and EMG recording in the LC involved three stages: wake, maintenance, and emergence. Corresponding to these three stages, the first 3 min after recording, 3 min before gaseous anesthesia by isoflurane cessation, and 3 min after 1 min of stimulation window (less than 1 min after tongue pinching by a clip or without a tongue pinch) were selected for BSR analysis in MATLAB and power analysis in NeuroExplorer 5. In studies involving anesthesia, BSR helps evaluate the depth of anesthesia. Higher BSR values indicate deeper levels of anesthesia, often associated with more profound neural suppression (*70*).

### Immunohistochemistry, imaging, and image analysis

Mice were deeply anesthetized with isoflurane (~15 s) and then perfused with saline for 4 min and 4% ice-cold paraformaldehyde solution for 3 min. After perfusion, the mouse brains were carefully removed and immersed in 4% paraformaldehyde solution overnight, followed by gradient dehydration sucrose solution (20 and 30%) at 4°C for dehydration to become isotonic. The brains were cut into 40-μm coronal slices at −20°C using a cryostat microtome system (CM1860, Leica). For immunofluorescence, the slices were washed three times with phosphate-buffered saline (PBS). After being blocked in the buffer (containing 0.5% Triton X-100, 5% donkey serum, and 5% bovine serum albumin (BSA) in PBS) for 1 hour at room temperature, the sections were incubated in buffers (containing 0.3% Triton X-100, 3% BSA, and 3% donkey serum in PBS) supplemented with primary antibodies, including anti-glutamate (1:500; rabbit, Sigma-Aldrich) and anti-GABA (1:500; rabbit, Sigma-Aldrich) at 4°C for 48 hours; anti–c-Fos (1:500; guinea pig, Synaptic Systems), anti–c-Fos (1:500; rabbit, Sigma-Aldrich), anti-TH (1:500; rabbit, Proteintech), anti-PV (1:500; rabbit, Abcam), anti-somatostatin (1:50; mouse, Santa Cruz Biotechnology), and anti-TUBB3 (1:500; Rabbit, Merck) at 4°C for 24 hours. Then, the slices were washed three times with PBS and incubated with the secondary antibodies (containing 0.1% Triton X-100, 1% BSA in PBS, and 0.2% secondary antibodies) at room temperature in the dark for 1.5 hours. Cell nuclei were stained with 4′,6-diamidino-2-phenylindole (1:1000; catalog no. D9542, Sigma-Aldrich) for 5 min. The fluorescence signals were imaged using an Olympus FV3000 microscope. Further analyses, such as analysis of cell counts and colocalization, were conducted using ImageJ software (Fiji edition, National Institutes of Health).

### Stereotaxic surgery and virus injection

Mice were deeply anesthetized with pentobarbital (20 mg/kg, i.p.) and were immobilized on a stereotaxic frame (RWD Life Science Inc., China). Then, sterile ointment was applied to each eye, and a miniature heating pad was placed under the mouse’s body to maintain the temperature at 37°C. After simple disinfection and a midline scalp incision, the skull surface was exposed with a midline scalp incision and its position was leveled. A syringe tip and adjustable speed dental drill (B67275, Meisinger, Germany) were used to open a small (~0.5 mm) craniotomy. Then, the virus was injected into the brain region specifically at a speed of 30 nl/min by using a 10-μl microsyringe (Gaoge) assembled with a calibrated glass microelectrode (1B 100-3, WPI, USA). Following the injection, the microsyringe was left in the injection site for 5 to 10 min to minimize virus leakage in the track. Last, the incision was sutured, and the surgical wound was sterilized with sterile ointment.

The injections were performed using the following stereotaxic coordinates: the LC coordinates: AP, −5.40 mm; ML, −0.89 mm; dorsal/ventraland (DV), −3.65 mm; the MTN coordinates: AP, −5.40 mm; ML, ±1.00 mm; DV, −3.50 mm; the NAcSh coordinates: AP, +1.10 mm; ML, ±0.58 mm; DV, −4.10 mm; the PVT coordinates: AP, −0.34 mm; ML, ±0.50 mm; DV, −3.55 mm, at an 8° lateral angle.

For anterograde tracing of the PVT → NAcSh circuit, rAAV2/1-hSyn-Cre-EGFP (1.04 × 10^13^ vg/ml, 200 nl, BrainVTA) was injected into the PVT, and rAAV2/9-DIO-mCherry (4.82 × 10^12^ vg/ml, 150 nl, BrainVTA) was injected into the NAcSh of C57 mice. Three weeks later, the EGFP and mCherry signals were costained with the glutamate-specific or GABA-specific antibodies to identify the cell types in the PVT or the NAcSh.

For anterograde tracing of the tongue → MTN circuit, rAAV2/9-DIO-mCherry-mCherry (5.57 × 10^12^ vg/ml, 150 nl, BrainVTA) was injected into the MTN of *VGluT2-Cre* mice. For retrograde monosynaptic tracing of the tongue → MTN circuit, a rAAV2/Retro-hSyn-Cre (5.13 × 10^12^ vg/ml, 2 μl, BrainVTA) virus was injected into the tongue, which could be absorbed by the terminals at the injection site and transported retrogradely to the soma to express Cre recombinase, and a Cre-dependent rAAV2/9-DIO-EGFP (5.34 × 10^12^ vg/ml, 150 nl, BrainVTA) virus was bilaterally injected into the MTN of C57 mice. For retrograde transsynaptic tracing of the tongue, PRV-CAG-EGFP (2.00 ×10^09^ PFU/ml, 2 μl, BrainVTA) was injected into the tongue of C57 mice. One week later, the mice were perfused transcardially, and brain slices contain the MTN were stained with a glutamate-specific antibody.

For chemogenetic manipulation of the tongue → MTN circuit, a rAAV2/Retro-hSyn-Cre (5.13 × 10^12^ vg/ml, 2 μl) virus was injected into the tongue, and a Cre-dependent rAAV2/9-DIO-hM3Dq-mCherry (2.56 × 10^12^ vg/ml, 120 nl, BrainCase) or rAAV2/9-DIO-hM4Di-mCherry (2.41 × 10^12^ vg/ml, 120 nl, BrainVTA) virus was bilaterally injected into the MTN of C57 mice. For chemogenetic manipulation of the PVT → NAcSh circuit, an rAAV2/9-DIO-hM3Dq-mCherry (200 nl) or rAAV2/9-DIO-hM4Di-mCherry (200 nl) virus was injected into the PVT of *CaMKII-Cre* mice. Mice infused with the same volume of the rAAV2/9-DIO-mCherry virus into the MTN or PVT were used as controls. A catheter (diameter of 250 μm, double tube with a 1.00-mm interval and 4.20-mm depth, RWD Life Science Inc., China) was initially implanted into both side of the NAcSh of an anesthetized mouse that was immobilized in a stereotaxic apparatus, and the cannula was secured to the skull with dental cement.

### Chemogenetic manipulation

After three weeks of viral expression, the mice were transported to the testing room and were habituated for at least 1 hour before testing. For chemogenetic manipulation of the PVT → NAcSh circuit, the chemical ligand CNO (3 μM, 200 nl, Sigma-Aldrich, USA) or vehicle (dimethyl sulfoxide mixed with 0.9% NaCl) was infused at a rate of 10 nl/s into the NAcSh of isoflurane-anesthetized mice. Behavioral tests were then carried out at least 30 min later. For the control group, the same manipulation protocol was used, and a vehicle was delivered via implanted cannulas. For chemogenetic manipulation of the tongue → MTN circuit, the chemical ligand CNO (5 mg/kg) was intraperitoneally injected into these mice under isoflurane anesthesia. Behavioral tests were then carried out at least 30 min later. For the control group, the same manipulation protocol was used. After all behavioral tests were completed, the mice were euthanized to verify the location of the virus injection site and the catheter implantation site. The data obtained from mice that missed the target brain regions were excluded from our analysis.

### Tissue clarity and morphological reconstruction

The tongue and trigeminal ganglion were cleared using three-dimensional (3D) imaging of solvent-cleared organs with superior fluorescence-preserving capability (FDISCO) protocol. For tissue clearing, the dissected trigeminal ganglion and tongue were incubated in 4% PFA at 4°C with shaking overnight and washed twice with 1× PBS for ~2 hours at room temperature. After clearing, the fixed trigeminal ganglion and tongue samples were dehydrated with tetrahydrofuran solutions (mixed with dH_2_O, pH adjusted to 0.9 with triethylamine) at a series of concentrations of 50, 70, 80, and 100 vol % (twice or thrice). Pure dibenzyl ether (108014, Sigma-Aldrich) was used as a refractive index matching solution to clear the tissue after dehydration. All steps were performed at 6° to 8°C with slight shaking. During clearing, the tissues were placed in glass chambers covered with aluminum foil in the dark.

For tongue tissue, 3D fluorescence images of the cleared samples were obtained using a light sheet microscope (LiToneXL, Light Innovation Technology, China) equipped with a 4× objective lens [numerical aperture (NA) = 0.28], and thin light sheets were used to illuminate the four sides of the samples during imaging. For trigeminal ganglion imaging, the cleared samples were mounted on glass slides and imaged by an inverted confocal fluorescence microscope (LSM880, Zeiss, Germany) equipped with a 10× objective (NA = 0.5). To acquire images, the cleared samples were manually attached to the sample holder adapter. Subsequently, the samples were immersed in imaging reagent within a 3D printing sample chamber and excited with light sheets with a wavelength of 594 nm. The raw data (tiff. images) of the tongue were stitched and converted using LitScan software, while the collected trigeminal ganglion images were processed with ZEN software (Zeiss, Germany), and all the data were visualized by IMARIS software (Bitplane).

### Fiber photometry recording

To detect the calcium signals of PVT^Glu^ neurons, an rAAV2/Retro-hSyn-Cre virus was injected into the NAcSh, and a Cre-dependent rAAV2/9-DIO-GCaMP7s (2.00 × 10^12^ vg/ml, 150 nl, TailTool) virus was injected into the PVT, with an optic fiber implanted above the PVT of C57 mice. To detect the calcium signals of PVT-innervated NAcSh neurons, an anterograde ScAAV2/1-hSyn-Cre virus was injected into the PVT, and a Cre-dependent AAV-DIO-GCaMP7s virus was injected into the NAcSh, with an optic fiber implanted above the NAcSh of C57 mice. To detect the calcium signals of LC^NE^ neurons, a Cre-dependent rAAV2/9-DIO-GCaMP7s (150 nl) was injected into the LC, with an optic fiber implanted above the LC of *DBH-Cre* mice. The same strategy was applied to the *D1-Cre* and *D2-Cre* mice to detect the calcium signals of NACsh^D1-MSN^ and NACsh^D2-MSN^ neurons, respectively. To detect the changes in the calcium signals of LC^NE^ neurons when MTN^Glu^ neurons were ablated, a Flpo-dependent virus composed of rAAV2/9-fDIO-taCaspase3 (5.31 × 10^12^ vg/ml, 120 nl, BrainVTA) or ACSF of the same volume was bilaterally injected into the MTN, along with a rAAV2/Retro-hSyn-Flpo (5.31 × 10^12^ vg/ml, 2 μl, BrainVTA) injected into the tongue of *DBH-Cre* mice.

The fiber photometry recording was conducted 3 weeks after viral expression, and a fiber optic patch cord (Inper, MFO-1x2-F-W1.25-200-0.37-100) connected to the fiber photometry system (Inper) was attached to the implanted optic fiber using a ceramic sleeve with black heat-shrinkable tubes. To record fluorescence signals from GCaMP7s, light from a 470-nm light-emitting diode (LED) was bandpass-filtered (470/10 nm), collimated, reflected by dichroic mirrors (MD498, Thorlabs), coupled to an optical commutator (Doris Lenses) after being focused by a 20× objective lens (NA = 0.4, Olympus), and then delivered at a power of 25 to 40 μW at the tip of the fiber optic cannula to excite GCaMP7s fluorescence. Then, the fluorescence emitted from GCaMP7s was bandpass-filtered (525/40 nm, Thorlabs) and focused on the sensor of a complementary metal-oxide semiconductor camera. The end of the fiber was imaged at a frame rate of 40 fps with InperSignal, and the mean value of the region of interest (ROI) at the end face of the fiber was calculated using InperPlot software. To serve as an isosbestic control channel, 410-nm LED light was bandpass-filtered (410/10 nm) and delivered alternately with 470-nm LED light. GCaMP7s fluorescence intensity was then recorded in bystander mice and anesthetized mice, respectively. The values of fluorescence change (Δ*F/F*) were derived by calculating Δ*F/F* (%), which was calculated as (*F* – *F*_0_)/*F*_0_ × 100%, where *F*_0_ is the mean of the GCaMP7s signals in a 1-s-long period before the onset of the specific prosocial behaviors (sniffing, grooming, eye licking, and tongue dragging) of bystander mice. All heatmaps and averaged calcium traces with shaded areas denoting the SEM were generated in InperPlot software (Inper Technology, Hangzhou). Mice with missed targets of the optical fiber or failed virus injection were excluded after the examination.

### In vivo electrophysiology

Homemade movable tetrode arrays, equipped with screw-driven microdrives, were used to simultaneously record multi-tetrode electrical signals from multiple neurons. Each tetrode, composed of four twisted fine platinum/iridium wires (12.5-μm diameter, California Fine Wire, Grover Beach, CA), was implanted into the LC after 3 days, at which point the mice were housed in a single cage for environmental adaptation. The tetrodes were firmly affixed to the skull using four miniature skull screws, cyanoacrylate glue, and dental cement.

Mice were allowed to rest for at least 7 days to recover and were habituated to the cables connected to the electrodes on their heads before recording. Neurostudio software (Neurostudio, China) was used to record and process spiking activities, employing amplification, digitization at 40 kHz, and filtering within a 300- to 5000-Hz bandwidth. Then, the sorting of single-unit spikes was conducted using Offline Sorter software (Plexon Inc.) and Neuroexplorer 5 (Nex Technologies). The waveforms were first separated into individual clusters by principal components analysis (PCA) and an unsupervised clustering algorithm based on a κ-means method was used. Data analysis included only well-isolated units [*L* ratio < 0.2, isolation distance >15 and exhibit recognizable refractory periods (>2 ms) in the interspike interval histograms].

For in vivo optogenetic tagging of LC^NE^ neurons, rAAV2/9-DIO-ChR2-mCherry (5.54 × 10^12^ vg/ml, 150 nl, BrainVTA) virus was injected into the LC of *DBH-Cre* mice. Three weeks later, optrodes were implanted at the same coordinates where the virus was injected. The optrode was constructed by surrounding an optical fiber with tetrode wires, and the tip of the optical fiber was approximately 200 μm above the tetrode tips. Blue light pulses (470 nm, 2-ms duration, 20 Hz, 2 to 5 mW) were delivered at the end of each recording session at high frequencies. Units were considered light responsive if they exhibited time-locked spiking with high reliability (>90%), short first-spike latency (<3 ms), and low jitter (<2 ms) after light-pulse illumination. Only when the waveforms of laser-evoked and spontaneous spikes were highly similar (correlation coefficient > 0.9) were considered to originate from a single neuron.

### In vitro electrophysiological recordings

#### Acute brain slice preparation

Mice were anesthetized with pentobarbital (20 mg/kg, i.p.). After intracardial perfusion with ~20 ml of ice-cold *N*-methyl-d-glucamine (NMDG) ACSF containing the following 93 mM NMDG, 20 mM Hepes buffer, 25 mM glucose, 2.5 mM KCl, 0.5 mM CaCl_2_, 1.2 mM NaH_2_PO_4_, 30 mM NaHCO_3_, 10 mM MgSO_4_, 5 mM sodium ascorbate, 3 mM sodium pyruvate, 2 mM thiourea, and 3 mM glutathione (pH: 7.3 to 7.4, osmolarity: 300 to 305 mOsm/kg), coronal slices of the LC (300 μm) were sectioned in chilled (2° to 4°C) NMDG ACSF on a microtome (VT1200S, Leica, Germany) vibrating at velocity of 0.18 mm/s. After that, the brain slices were initially incubated in NMDG ACSF (saturated with 95% O_2_/5% CO_2_ to provide stable pH and continuous oxygenation) for ~12 min at 33°C, followed by recovery for at least 1 hour at 28°C in (Hepes) ACSF containing 92 mM NaCl, 2.5 mM KCl, 2 mM CaCl_2_, 1.2 mM NaH_2_PO_4_, 30 mM NaHCO_3_, 5 mM Na-ascorbate, 3 mM Na-pyruvate, 25 mM glucose, 20 mM Hepes, 2 mM MgSO_4_, 3 mM glutathione, and 2 mM thiourea (pH: 7.3 to 7.4, osmolarity: 300 to 310 mOsm/kg). The brain slices were transferred to a slice chamber (Warner Instruments, USA) for electrophysiological recording and were continuously perfused with standard ACSF that contained 129 mM NaCl, 2.4 mM CaCl_2_, 3 mM KCl, 1.3 mM MgSO_4_, 20 mM NaHCO_3_, 1.2 mM KH_2_PO_4_ and 10 mM glucose (pH: 7.3 to 7.4, osmolarity: 300 to 305 mOsm/kg) with a flow rate of 2.5 to 3 ml/min. The temperature of the standard ACSF was maintained at 32°C by an in-line solution heater (TC-344B, Warner Instruments, USA). During slice preparation and electrophysiology recording, all solutions were continuously bubbled with 95% O_2_/5% CO_2_.

#### Whole-cell patch-clamp recordings

Neurons were visualized with a 40× water-immersion objective on an infrared (IR)–differential interference contrast (DIC) microscope (BX51WI, Olympus, Japan), and an infrared camera was connected to the video monitor. Recording pipettes (3 to 5 megohm) were pulled from borosilicate glass capillaries (VitalSense Scientific Instruments Co., Ltd., Wuhan, China) with an outer diameter of 1.5 mm on a four-stage horizontal puller (P-1000, Sutter Instruments, USA). Whole-cell patch-clamp recordings were controlled by a MultiClamp 700B amplifier combined with low-pass filtering at 2.8 kHz and digitized at 10 kHz. To test the effects of CNO on neuronal excitability ex vivo, AAV-DIO-hM3Dq-mCherry virus was injected into the MTN and AAV2/Retro-hSyn-Cre injected into the tongue of C57 mice. Then, 3 to 4 weeks after injection, coronal slices containing the MTN were prepared, and mCherry^+^ neurons (indicative of the viral expression) were selected for whole-cell patch-clamp recordings. After 4 min of baseline recording for the rest membrane potential, 10 μM CNO was added into the ACSF, and the neurons were recorded at least 10 min in follow for observation of the effects of CNO. Neurons with series resistance of more than 30 megohm or that changed by more than 20% during the recording were excluded.

#### Light-evoked response

A laser (Shanghai Fiblaser Technology, China) through an optical fiber [200-mm outside diameter (OD), 0.37 NA, inper] was used to deliver optical stimulation with positioned 0.2 mm above the surface of the target brain region. To test the function of the PVT^Glu^ → NAcSh^PV^ pathway, AAV-CaMKII-ChR2-mCherry virus was injected into the PVT and AAV-DIO-EGFP virus was injected into the NAcSh of *PV-Cre* mice. Light-evoked postsynaptic currents were elicited by 20-ms blue laser light (473 nm, 20 Hz, 10 mW) stimulation of axonal terminals of PVT neurons infected with ChR2-mCherry that projects to the NAcSh. The EPSCs were recorded with the membrane potential held at −70 mV. For evaluating the function of the MTN^Glu^ → LC^NE^ pathway, a mixture of Flpo-dependent rAAV2/9-fDIO-ChR2-EGFP (5.23 × 10^12^ vg/ml, 100 nl, BrainVTA) and rAAV2/9-DIO-mCherry viruses was injected into the MTN (adjacent to the LC), along with a rAAV2/Retro-hSyn-Flpo (5.31 × 10^12^ vg/ml, 2 μl, BrainVTA) was injected into the tongue of *DBH-Cre* mice.

### Microendoscopic calcium imaging and data processing

For the microendoscopic experiments, 150 nl of mixed virus solution (ScAAV2/1-hSyn-Cre diluted 1:5 in rAAV2/9-DIO-GCaMP7s) was injected into the NAcSh of C57 mice. Then, a microendoscopic gradient index (GRIN) lens (6.1 mm in length, 0.5 mm in diameter, Inscopix) was implanted into the NAcSh (ML, −0.58 mm; AP, +1.10 mm; DV, −4.10 mm), and the lenses were secured to the skull with dental cement and skull nails. After surgery, the mice were allowed to recover for at least 3 weeks. Mice were acclimated to the weight of the microscope and the experimental box daily for 15 min sessions. Before data acquisition, the mice were briefly anesthetized with isoflurane, and an integrated miniature fluorescence microscope (Inscopix) was attached to the GRIN lens. During each trial of behavioral testing, imaging data and behavior were simultaneously recorded at 20 fps for 15 min with a fluorescence power of 0.6 mW/mm^2^ (475 nm) and a resolution of 1280 × 800 pixels, with the same parameters of focus and position of the microendoscopic imaging to ensure the same field of view (FOV) over time. The microscope was detached from the baseplate, and the mouse was returned to its home cage after completion of the imaging sessions. All recordings were performed between 09:00 and 10:00 in the experimental box, which is consistent between groups.

For data analysis, fluorescence videos were processed offline by Inscopix data processing software (v.1.3.1) based on principal and independent component analysis. Candidate cells were manually selected on the basis of the Δ*F/F* and cell morphology in the ROI to exclude false-positive neurons. In general, data from three to five behavioral events were selected from each rescue-like behavior testing session. According to the difference in the mean Ca^2+^ fluorescence intensity among neurons, all the traces under the same experimental conditions were aligned and sorted. The traces were obtained for individual neurons by calculating the temporal *z* scores from −1 to 3 s, where 0 denoted the onset of each behavior. The *z* scores were computed as (*X*_(*t*)_ – *X*_m_)/SD, where *X*_(*t*)_ is the Δ*F*/*F* value at time *t* and *X*_m_ and SD are the mean of Δ*F*/*F* and SD of the Δ*F*/*F* values over a baseline period of −1 to 0 s, respectively.

To investigate the changes in neuronal activity, the pooled traces were averaged, and the overall activity difference between the 3-s post-response average and the 1-s pre-response average was calculated for each individual neuron. To classify the neurons based on the response type, the activity difference of individual neurons was compared with a threshold of the SD of the overall neuronal activity difference within the trial. If the activity difference was greater than the threshold, then the neuron was considered to be an excited cell; if the activity difference was less than the threshold, then the neuron was considered to be an inhibited cell; otherwise, the neuron was classified as a nonresponsive cell. The proportion of neurons in each group was then calculated.

### Statistical analysis

OriginPro 2017 software (OriginLab Corporation, USA) and GraphPad Prism 8 (GraphPad Software, Inc., USA) were used for the statistical analyses and graphing. Offline analysis of the data obtained from electrophysiological recordings was conducted using Clampfit software version 10.7 (Axon Instruments Inc., USA). The Shapiro-Wilk normality test was used for conformity with Gaussian-distributed residuals before *t* tests, analysis of variance (ANOVA), and descriptive statistics. We conducted statistical comparisons between two groups using paired or unpaired Student’s *t* tests. One-way or two-way ANOVA and Bonferroni post hoc analyses were used for analyses of multiple experimental groups. Nonparametric tests were conducted when data are not normally distributed, including Wilcoxon matched-pairs signed-rank test, Mann-Whitney test, and Kruskal-Wallis test. Data are shown as individual values or expressed as the means ± SEMs, and the significance levels are indicated as *P < 0.05, **P < 0.01, ***P < 0.001, and not significant (n.s.). *P* values less than 0.0001 are not provided as exact values. All the statistical tests, significance analyses, number of individual experiments, and other relevant information for data comparison are specified in table S1.
