# Supplementary material for: Rescue-like behavior in a bystander mouse toward anesthetized conspecifics promotes arousal via a tongue-brain connection
Source: Sci Adv. 2025 Jan 22;11(4):eadq3874. doi: 10.1126/sciadv.adq3874 (PMC11753405; doi:10.1126/sciadv.adq3874)
Supplement: Supplementary file 1 — Figs. S1 to S15 Tables S1 and S2 Legends for movies S1 to S5 [file sciadv.adq3874_sm.pdf]

Science Advances
AAAS

Supplementary Materials for

Rescue-like behavior in a bystander mouse toward anesthetized conspecifics promotes arousal via a tongue-brain connection

Peng Cao *et al.*

Corresponding author: Wenjuan Tao, wjtao01@ahmu.edu.cn;
Likui Wang, wlk9560@163.com; Zhi Zhang, zhizhang@ustc.edu.cn

Sci. Adv. **11**, eadq3874 (2025)
DOI: 10.1126/sciadv.adq3874

The PDF file includes:

Figs. S1 to S15
Tables S1 and S2
Legends for movies S1 to S5

Other Supplementary Material for this manuscript includes the following:

Movies S1 to S5

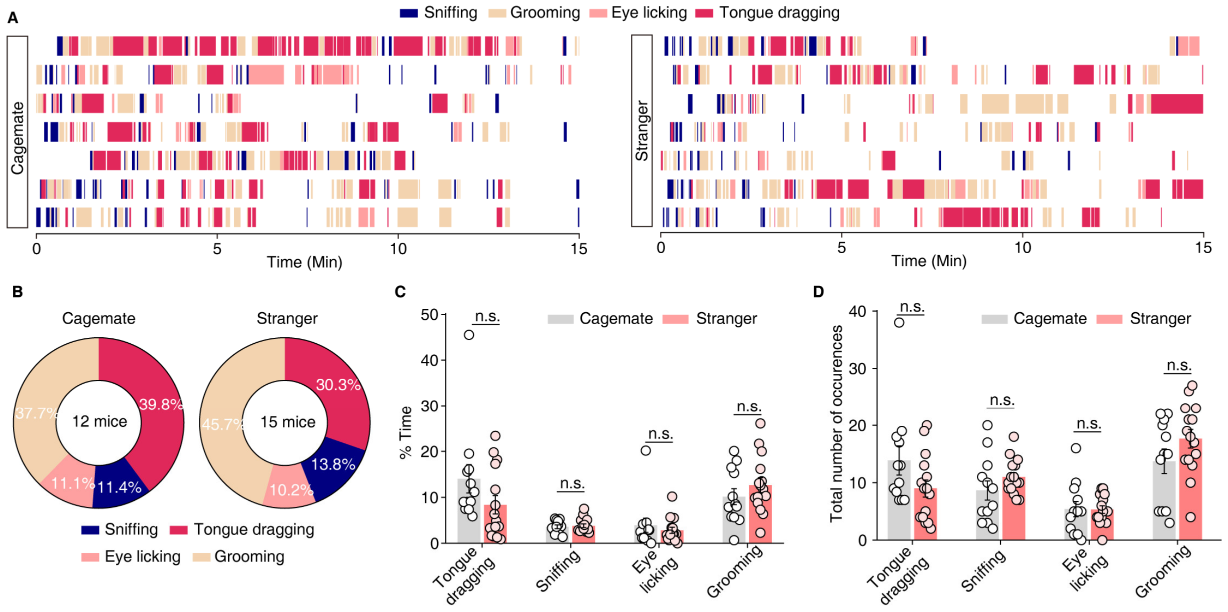

Fig. S1. Prosocial interactions of bystander mice with anesthetized cagemates and stranger mice. (**A**) Example raster plots showing tongue dragging, sniffing, eye licking, and grooming of bystander mice during prosocial interactions with their cagemates (left) or stranger mice (right). (**B**) The proportion of various behaviors in bystander mice toward cagemates (left) or stranger mice (right). (**C** and **D**) Total duration percentage (C) and the number of occurrences (D) for various behaviors in bystander mice. N (cagemate) = 12 mice; N (stranger) = 15 mice. Data are presented as the mean ± SEMs. n.s., not significant. Details of the statistical analyses are presented in Table S1.

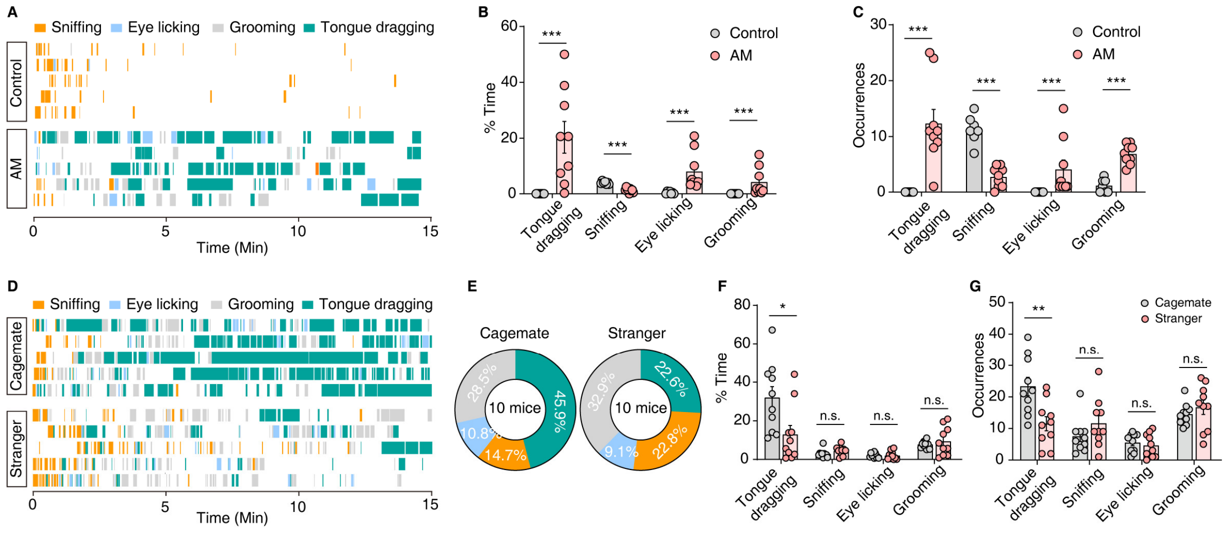

Fig. S2. Prosocial interactions of female bystander mice with anesthetized cagemates and stranger mice. (**A**) Example raster plots showing tongue dragging, sniffing, eye licking, and grooming in female bystander mice during prosocial interactions with anesthetized mice (AM). (**B** and **C**) Total duration percentage (B) and the number of occurrences (C) for various behaviors of female bystander mice toward saline-treated control mice or AM. N (control) = 7 mice; N (AM) = 9 mice. (**D**) Example raster plots showing tongue dragging, sniffing, eye licking, and grooming of female bystander mice during prosocial interactions with their anesthetized cagemates or stranger mice. (**E**) Proportion of various behaviors in female bystander mice toward cagemates (left) or stranger mice (right). (**F** and **G**) Total duration percentage (F) and the number of occurrences (G) for various behaviors in female bystander mice. N = 10 mice per group. Data are presented as the mean ± SEMs. * $P < 0.05$, ** $P < 0.01$, *** $P < 0.001$; n.s., not significant. Details of the statistical analyses are presented in Table S1.

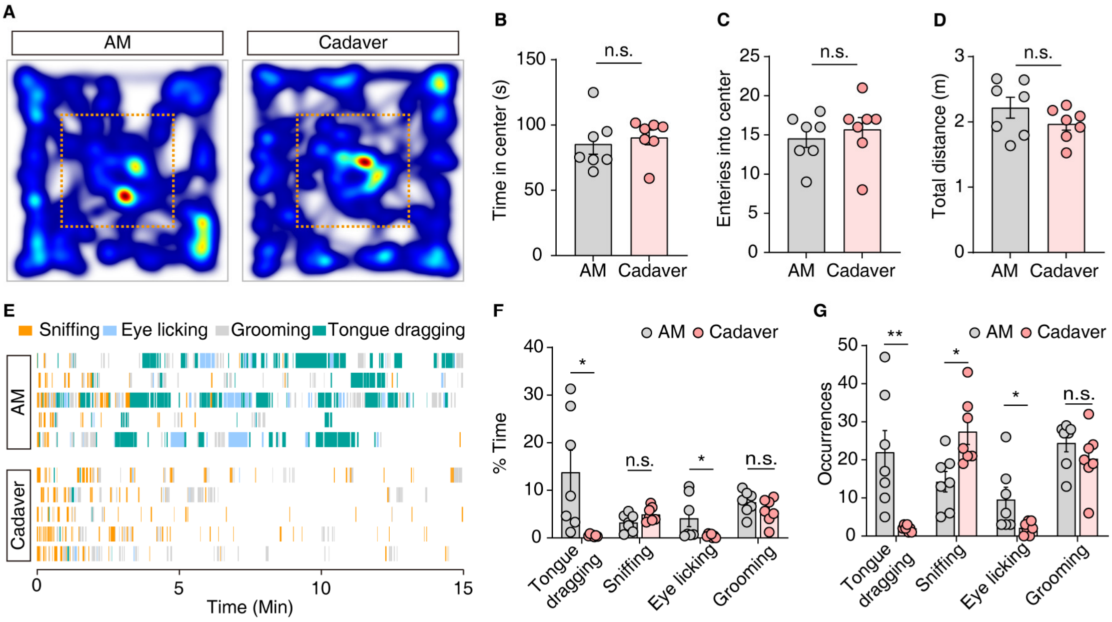

Fig. S3. Differences in rescue-like behaviors of bystander mice toward anesthetized mice and cadavers. (**A**) Representative heatmaps showing the locations of bystander mice paired with anesthetized mouse (AM) or a cadaver (yellow dotted circle) in the open field test (OFT). (**B** to **D**) Summary data for time spent (B), entries into the central area (C), and total distance (D) of the OFT from the indicated groups. N = 7 mice per group. (**E**) Example raster plots showing tongue dragging, sniffing, eye licking, and grooming of bystander mice during prosocial interactions with AM or cadaver. (**F** and **G**) Total duration percentage (F) and the number of occurrences (G) for various behaviors of bystander mice toward AM or cadaver. N = 7 mice per group. Data are presented as the mean ± SEMs. * $P < 0.05$, ** $P < 0.01$; n.s., not significant. Details of the statistical analyses are presented in Table S1.

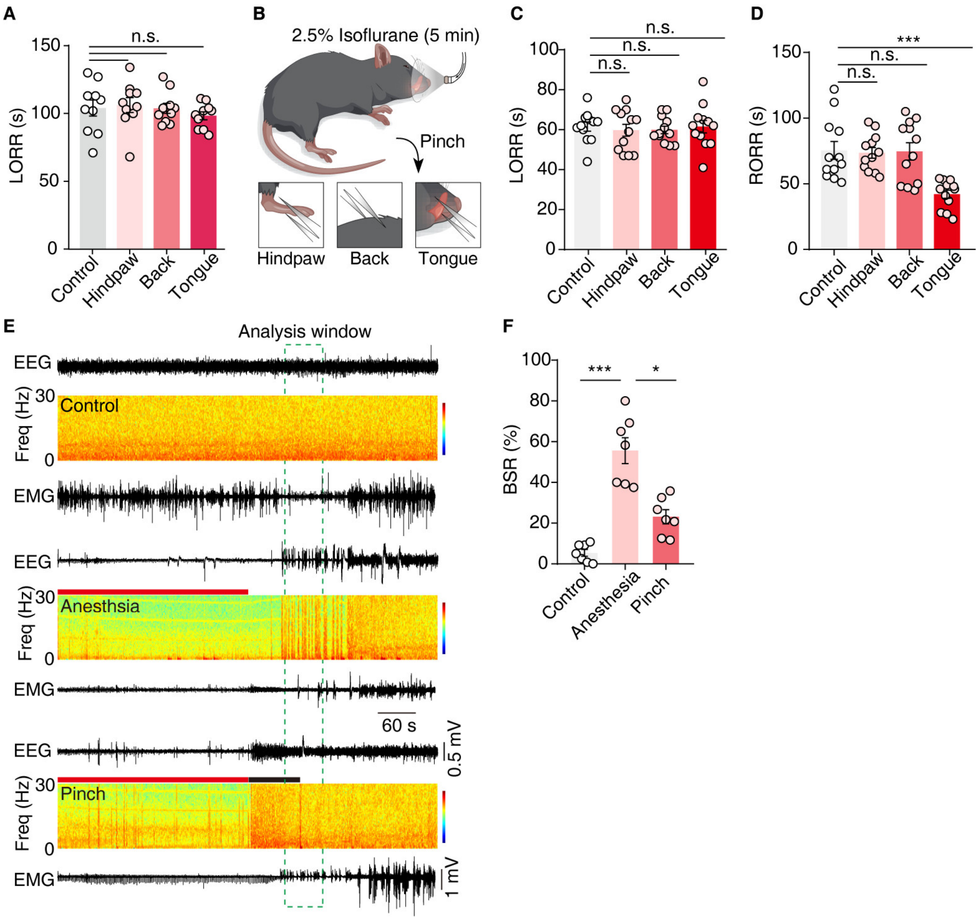

Fig. S4. Tongue pinching promotes arousal in anesthetized mice. (**A**) Summary data for anesthetic recovery (RORR, recovery of righting reflex) from 1.25% isoflurane-anesthetized mice after pinching the hindpaw, back, or tongue. N = 10 mice per group. (**B**) Schematic for the application of pinch stimuli to the hindpaw, back, or tongue of 2.5% isoflurane-anesthetized mice. (**C** and **D**) Summary data for LORR (C) and RORR (D) from 2.5% isoflurane-anesthetized mice after pinching the hindpaw, back, or tongue. N = 12 mice per group. (**E**) Examples of EEG and EMG data from awake control mice and 2.5% isoflurane-anesthetized mice treated with (pinch) or without tongue pinching (anesthesia) during the emergence period. Continuous EEG spectrograms are plotted below the raw EEG traces. (**F**) Summary data for the burst suppression ratio (BSR) from the analysis window indicated in (E) in the control, anesthesia, and pinch groups. N = 7 mice. Data are presented as the mean ± SEMs. * $P < 0.05$, *** $P < 0.001$; n.s., not significant. Details of the statistical analyses are presented in Table S1.

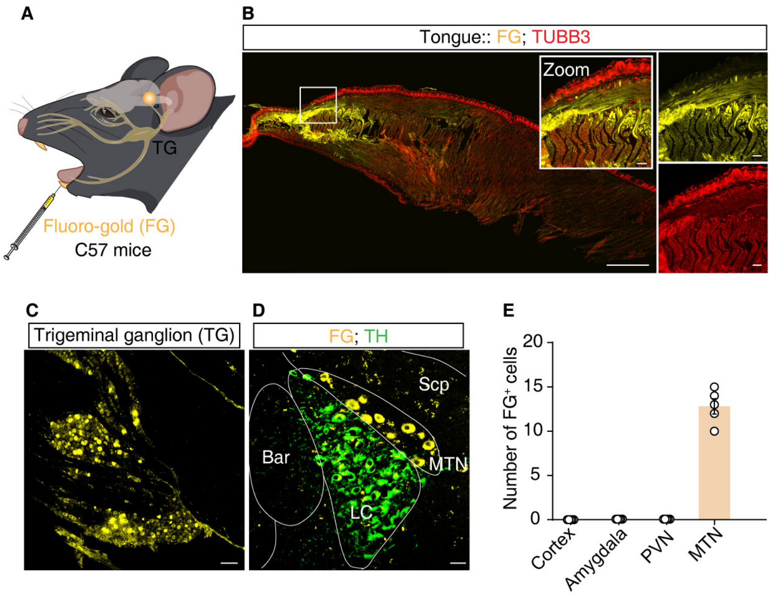

Fig. S5. Retrograde monosynaptic tracing from the tongue. (**A**) Schematic of Fluoro-Gold (FG) injection into the tongue of C57 mice. (**B**) Representative images of the injection site of FG in the tongue. Scale bar, 1000 μm. The inset depicts the area shown in the white box, in which FG^+ signals colocalized with the TUBB3 antibody (bottom). Scale bars, 50 μm. (**C** and **D**) Representative images showing FG^+ neurons in the trigeminal ganglion (C) and the MTN (D). Scale bars, 100 μm (TG) and 20 μm (MTN). (**E**) Summarized data showing the number of FG-labeled neurons in each brain region that projected to the tongue. MTN: mesencephalic trigeminal nucleus; LC: locus coeruleus; PVN: paraventricular nucleus. N = 5 mice. Data are presented as the mean ± SEMs.

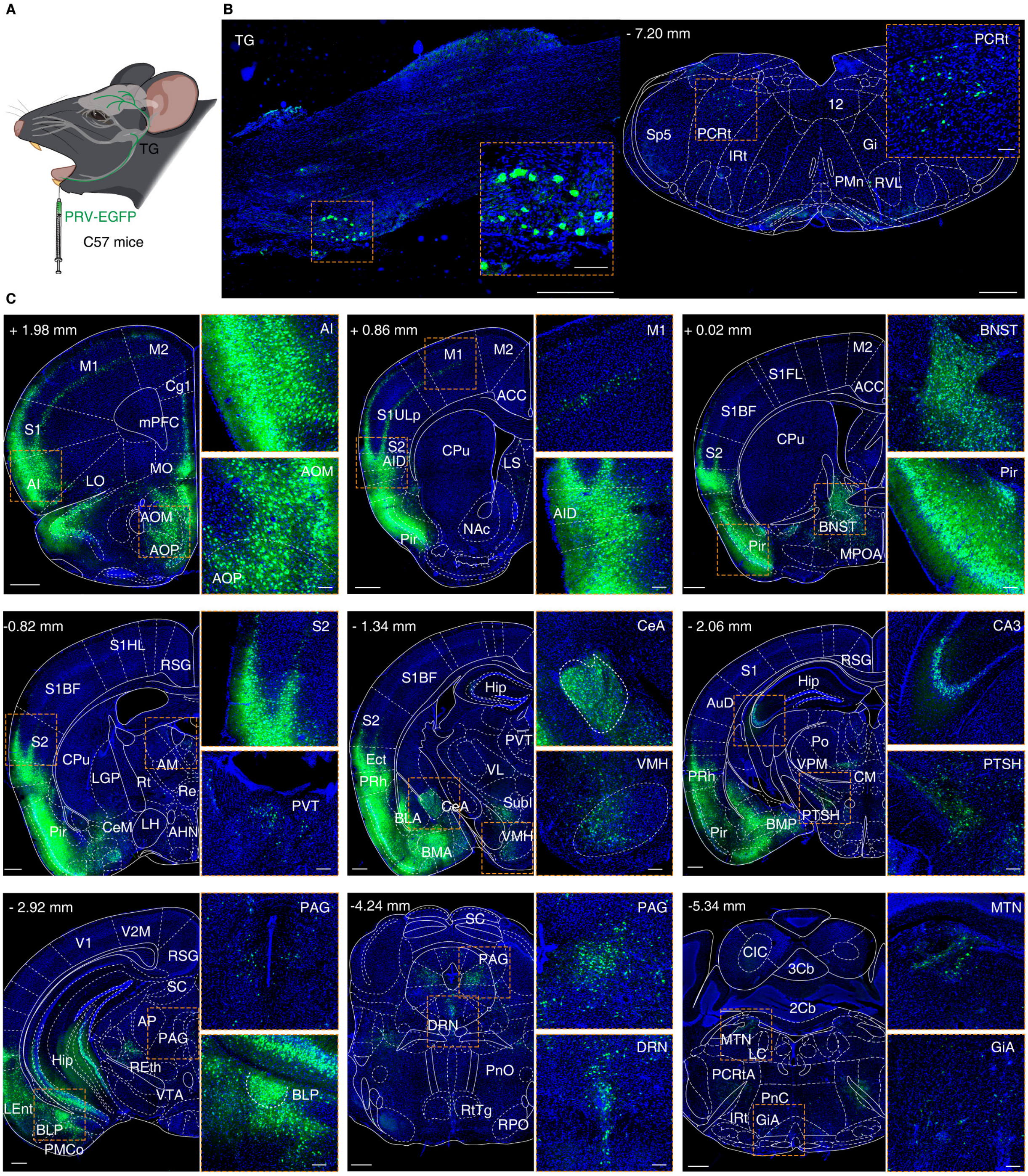

Fig. S6. Retrograde transsynaptic tracing from the tongue. (**A**) Schematic of retrograde transsynaptic tracing from the tongue using PRV-EGFP. (**B** and **C**) Representative images showing the distributions of $EGFP^+$ neurons traced from the tongue, including the trigeminal ganglion (TG) and multiple other brain regions. PCRt: parvicellular reticular nucleus; AI: agranular insular cortex; AOM: anterior olfactory nucleus; AOP: anterior olfactory area, posterior; M1: primary motor

cortex; AID: agranular insular cortex, dorsal part; Pir: piriform cortex; S2: secondary somatosensory cortex; BNST: bed nucleus of the stria terminalis; PVT: paraventricular thalamic nucleus; CeA: central amygdala; PRh: perirhinal cortex; VMH: ventromedial hypothalamic nucleus; SubI: subincertal nucleus; BLP: basolateral amygdaloid nucleus, posterior part; LEnt: lateral entorhinal cortex; PAG: periaqueductal gray; DRN: dorsal raphe nucleus; STH: subthalamic nucleus; CA3: field CA3 of the hippocampus; MTN: mesencephalic trigeminal nucleus; GiA: gigantocellular reticular nucleus, alpha. Scale bars, 500 μm (overview) and 100 μm (zoom).

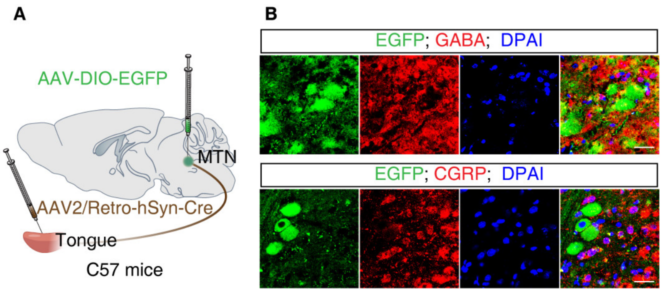

Fig. S7. Identification of tongue-projecting MTN^{Glu} neurons. (**A**) Schematic for anterograde monosynaptic tracing from the tongue to the MTN. (**B**) Representative images showing the EGFP-labeled tongue-projecting MTN neurons stained with GABA- and CGRP-specific antibodies. Scale bars, 20 μm.

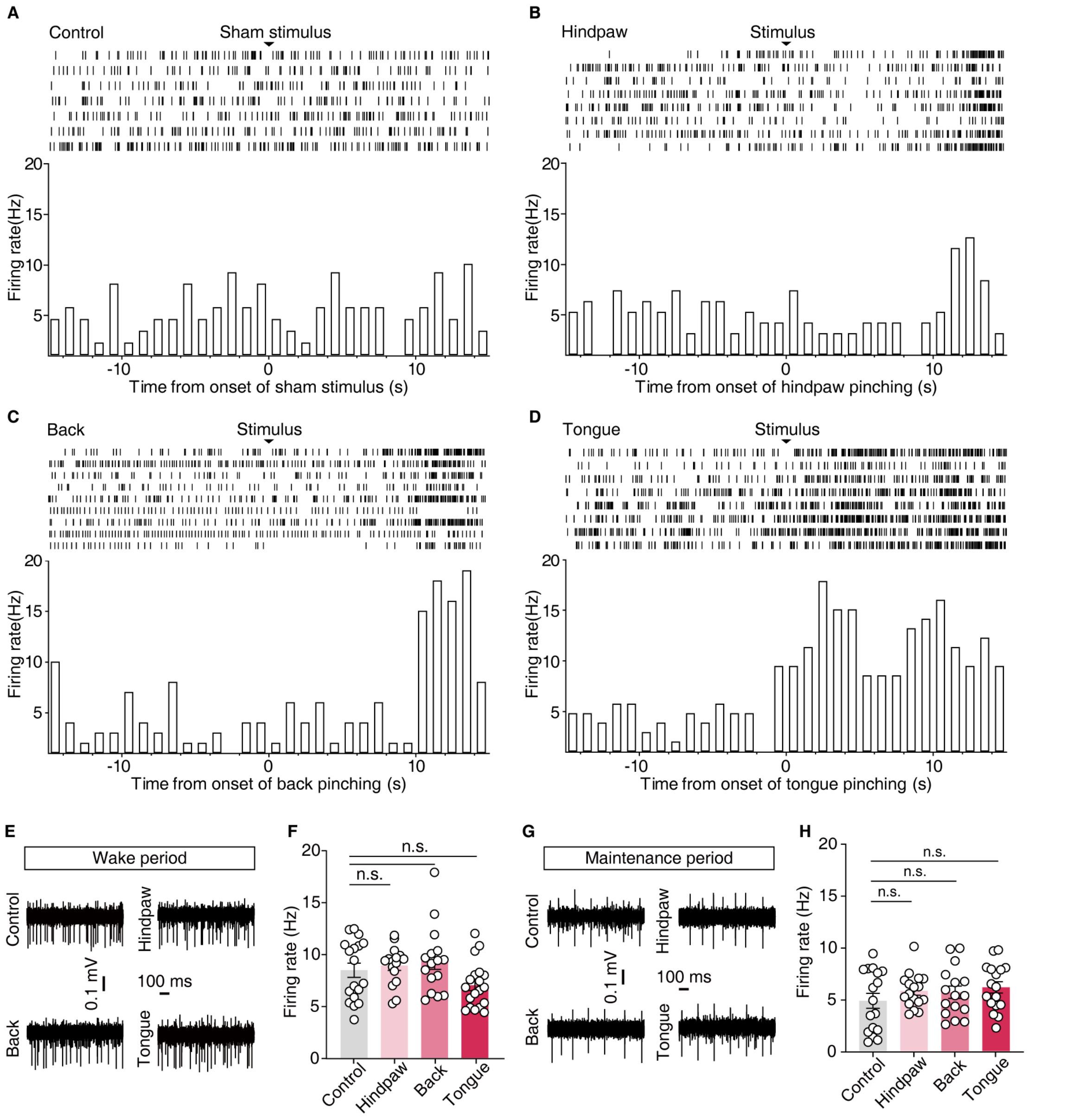

Fig. S8. Response of LC^{NE} neurons to pinching of different body parts. (**A**) Raster plot (top) and peri-stimulus time histogram (PSTH, bottom) showing LC^{NE} neuronal firing rates in anesthetized mice without pinching (control) during the emergence period. (**B** to **D**) As indicated in panel A, raster plots and PSTHs from pinched hindpaw (B), back skin (C), and tongue (D) during the emergence period. (**E** to **H**) Raster plots with typical traces (E, G) and quantitative data (F, H) of spontaneous firing rates of LC^{NE} neurons in anesthetized mice with or without (control) hindpaw, back, and tongue pinching during the wake (E and F) (N = 18 units from 4 mice for control; N = 16 units from 4 mice for hindpaw and back; N = 19 units from 4 mice for tongue) and

maintenance (G and H) (N = 17 units from 4 mice for control, hindpaw, and tongue; N = 16 units from 4 mice for back) periods. Data are presented as the mean ± SEMs. n.s., not significant. Details of the statistical analyses are presented in Table S1.

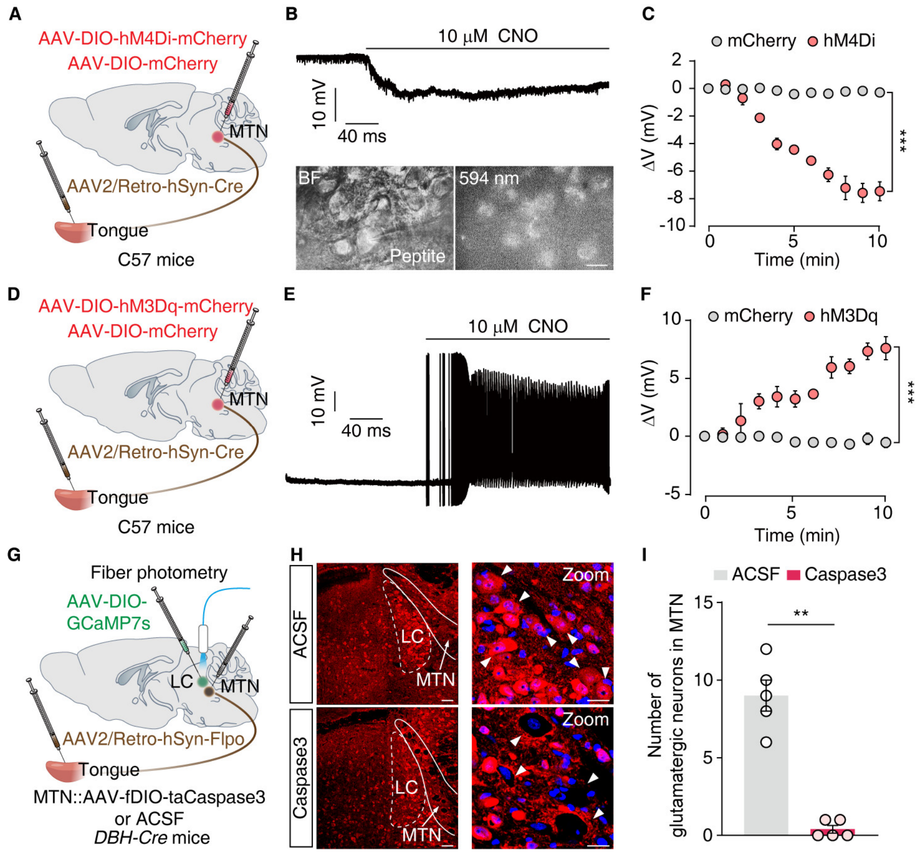

Fig. S9. Modulation of tongue-projecting MTN neuronal activity. (**A**) Schematic of chemogenetic inhibition of tongue-projecting MTN neurons. (**B**) A sample trace showing hyperpolarization of hM4Di-expressing MTN neurons after bath application with CNO diffusion (top); representative images showing whole-cell patch-clamp recordings and view of fluorescent channel (bottom). Scale bars, 20 μm. (**C**) Statistical data showing the average voltage magnitude of hyperpolarization recorded from hM4Di-expressing MTN neurons. N = 4 cells from 4 mice per group. (**D**) Schematic of chemogenetic activation of tongue-projecting MTN neurons. (**E** and **F**) A sample trace (E) and statistical data (F) from whole-cell patch-clamp recordings showing the average voltage magnitude of depolarization recorded from hM3Dq-expressing MTN neurons. N = 4 cells from 4 mice per group. (**G**) Schematic of virus injection and fiber photometry recordings *in vivo* in *DBH-Cre* mice. (**H** and **I**) Representative images (H) and statistical data (I) showing glutamate-positive neurons in the MTN and LC of ACSF- and taCaspase3-treated mice. N = 5 mice per group. Scale bars, 50 μm (overview) and 20 μm (zoom). Data are presented as the mean

± SEMs. ** $P < 0.01$, *** $P < 0.001$; n.s., not significant. Details of the statistical analyses are presented in Table S1.

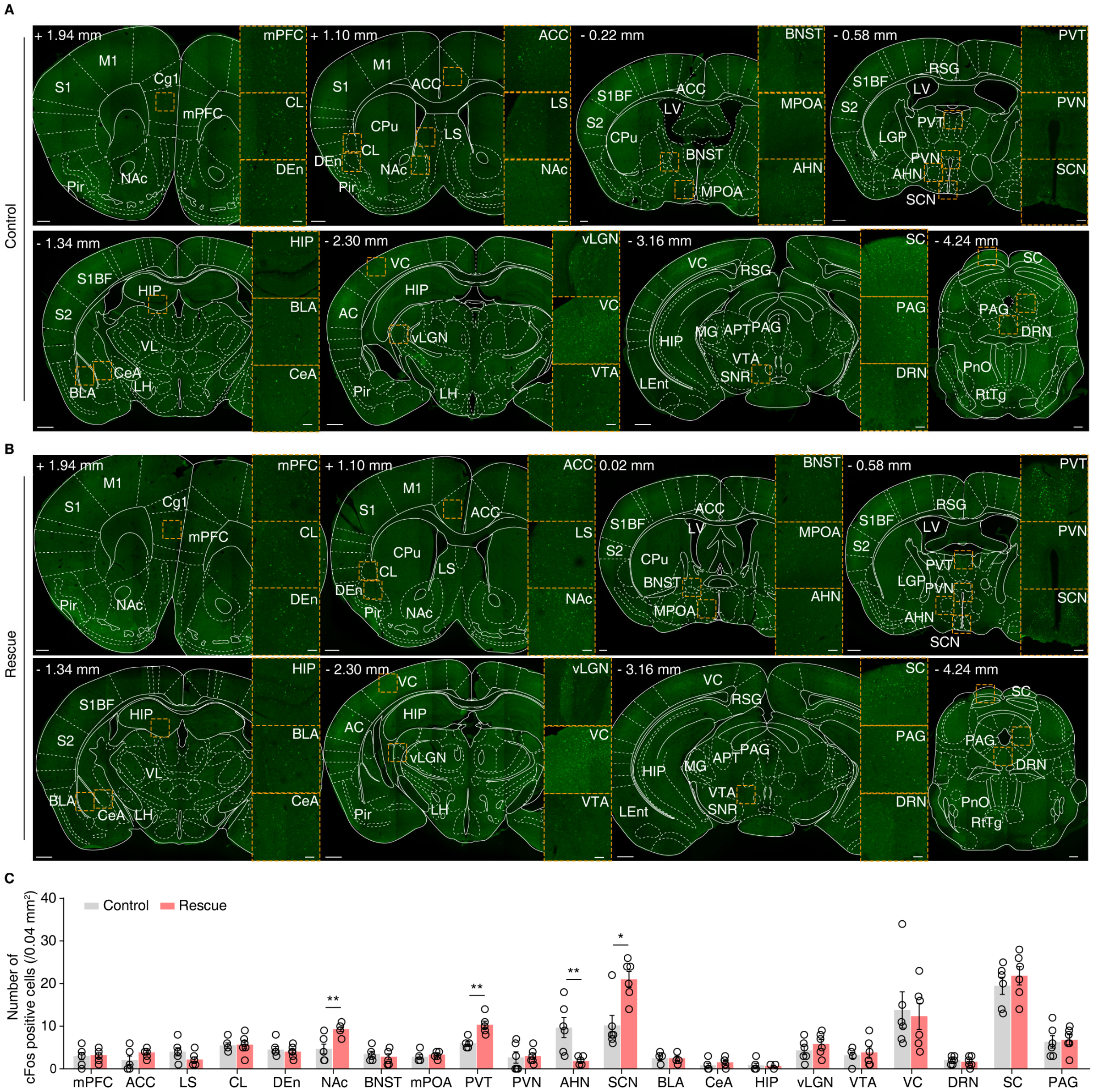

Fig. S10. c-Fos expression in bystander mice after displayed rescue-like behavior. (**A** and **B**) Representative images showing the distribution of c-Fos^{+} neurons in multiple brain regions of bystander mice in the absence (control, A) or presence (rescue, B) of anesthetized cagemate. Scale bars, 500 μm (overview) and 100 μm (zoom). (**C**) Number of c-Fos^{+} neurons per 0.04 mm^2 imaging area, including the medial prefrontal cortex (mPFC), anterior cingulate cortex (ACC), lateral septal nucleus (LS), claustrum (CL), dorsal endopiriform nucleus (DEn), nucleus accumbens (NAc), medial preoptic area (mPOA), paraventricular nucleus of the thalamus (PVT), anterior hypothalamic nucleus (AHN), suprachiasmatic nucleus (SCN), basolateral amygdala (BLA),

hippocampus (HPC), ventrolateral geniculate nucleus (vLGN), ventral tegmental area (VTA), visual cortex (VC), superior colliculus (SC), and periaqueductal gray (PAG). N = 6 mice. Data are presented as the mean ± SEMs. * $P < 0.05$, ** $P < 0.01$. Details of the statistical analyses are presented in Table S1.

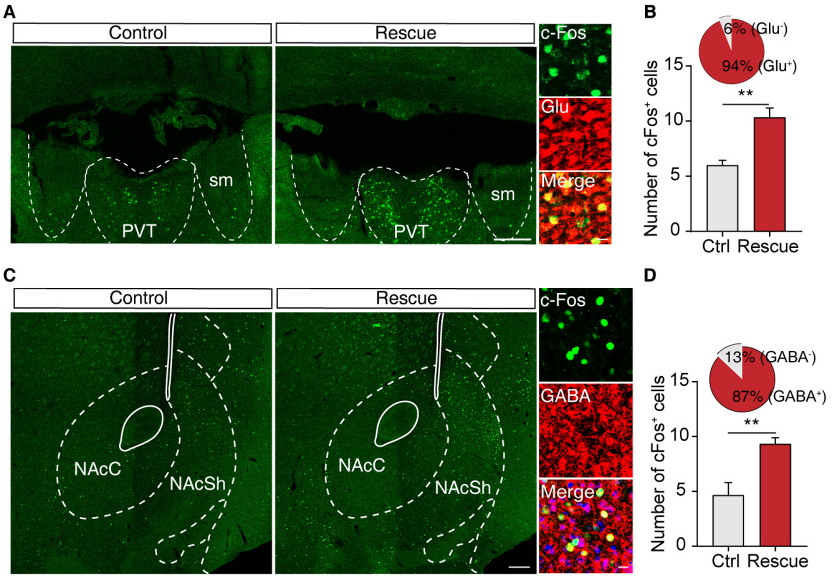

Fig. S11. c-Fos expression in the NAcSh and the PVT of bystander mice. (**A** and **B**) Representative images (A) and statistical data (B) showing that c-Fos$^+$ neurons within the PVT of bystander mice in the absence (control) or presence (rescue) of an anesthetized cagemate. The pie chart indicates that c-Fos$^+$ signals were mainly colocalized with a glutamate-specific antibody. Scale bars, 200 µm (overview) and 20 µm (inset). N = 6 mice. (**C** and **D**) As indicated in panels A and B, representative images (C) and statistical data (D) of the NAcSh of bystander mice. Scale bars, 200 µm (overview) and 20 µm (inset). N = 6 mice. Data are presented as the mean ± SEMs. ** $P < 0.01$. Details of the statistical analyses are presented in Table S1.

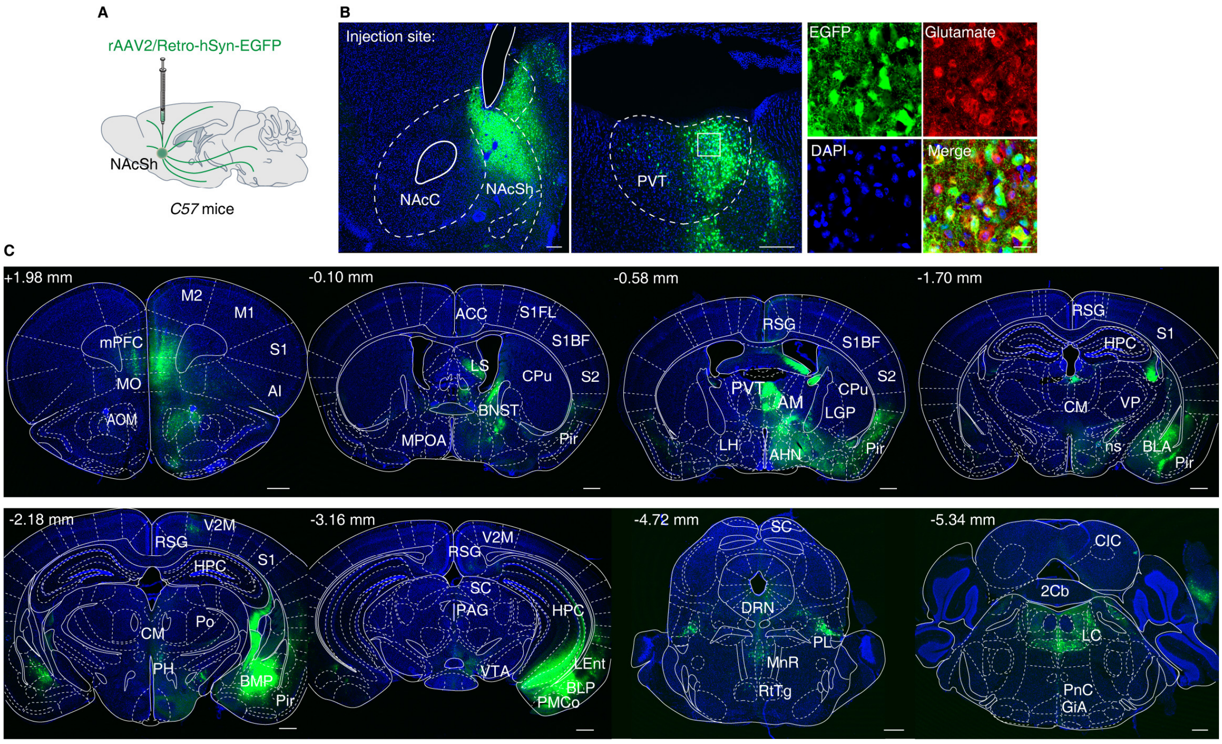

Fig. S12. Inputs of NAcSh neurons. (**A**) Schematic of retroAAV-hSyn-EGFP virus injection into the NAcSh of C57 mice. (**B**) Representative images of the injection site of retroAAV-hSyn-EGFP in the NAcSh and traced EGFP$^+$ neurons in the PVT (left). Scale bars, 200 µm. The inset depicts the area shown in the white box, in which EGFP$^+$ neurons colocalized with a glutamate-specific antibody (right). Scale bar, 20 µm. (**C**) Representative images showing EGFP$^+$ neurons in the mPFC, AOM, LS, BNST, PVT, AHN, lateral hypothalamus (LH), VTA, medial part of the secondary visual cortex (V2M), HPC, LEnt, BLA, DRN, paralemniscal nucleus (PL), median raphe nucleus (MnR), and LC, respectively. Scale bars, 500 µm.

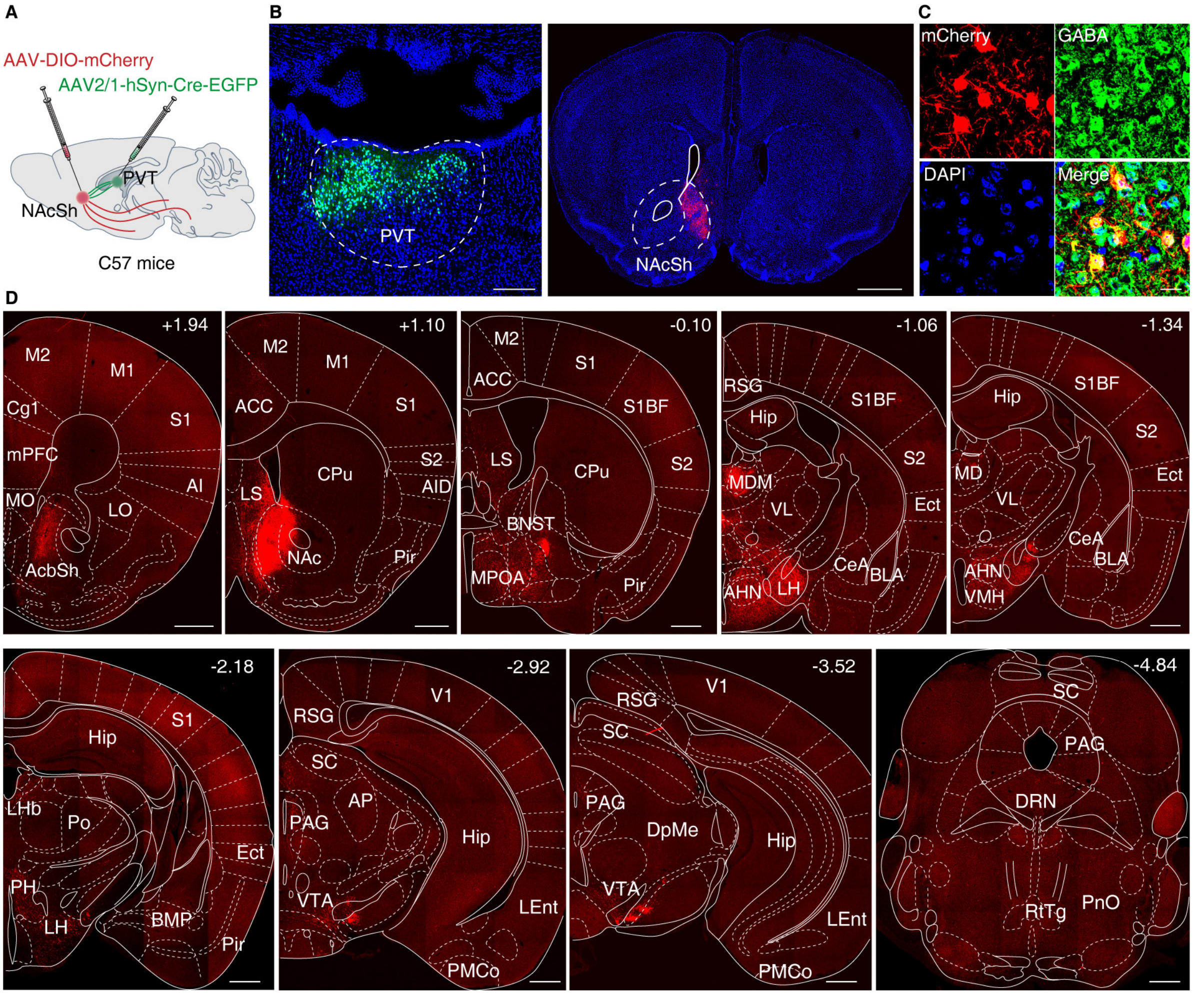

Fig. S13. Outputs of PVT-innervated NAcSh neurons. (**A**) Schematic for anterograde monosynaptic tracing from the PVT to the NAcSh. (**B**) Representative images showing viral expression in the PVT and NAcSh of C57 mice. Scale bars, 200 μm (PVT) and 1000 μm (NAcSh). (**C**) Typical images showing the mCherry$^+$ neurons in the NAcSh traced from the PVT were colocalized with a GABA-specific antibody. Scale bar, 20 μm. (**D**) Representative images showing the distributions of mCherry$^+$ terminals originating from PVT-innervated NAcSh neurons in multiple brain regions. Scale bars, 500 μm.

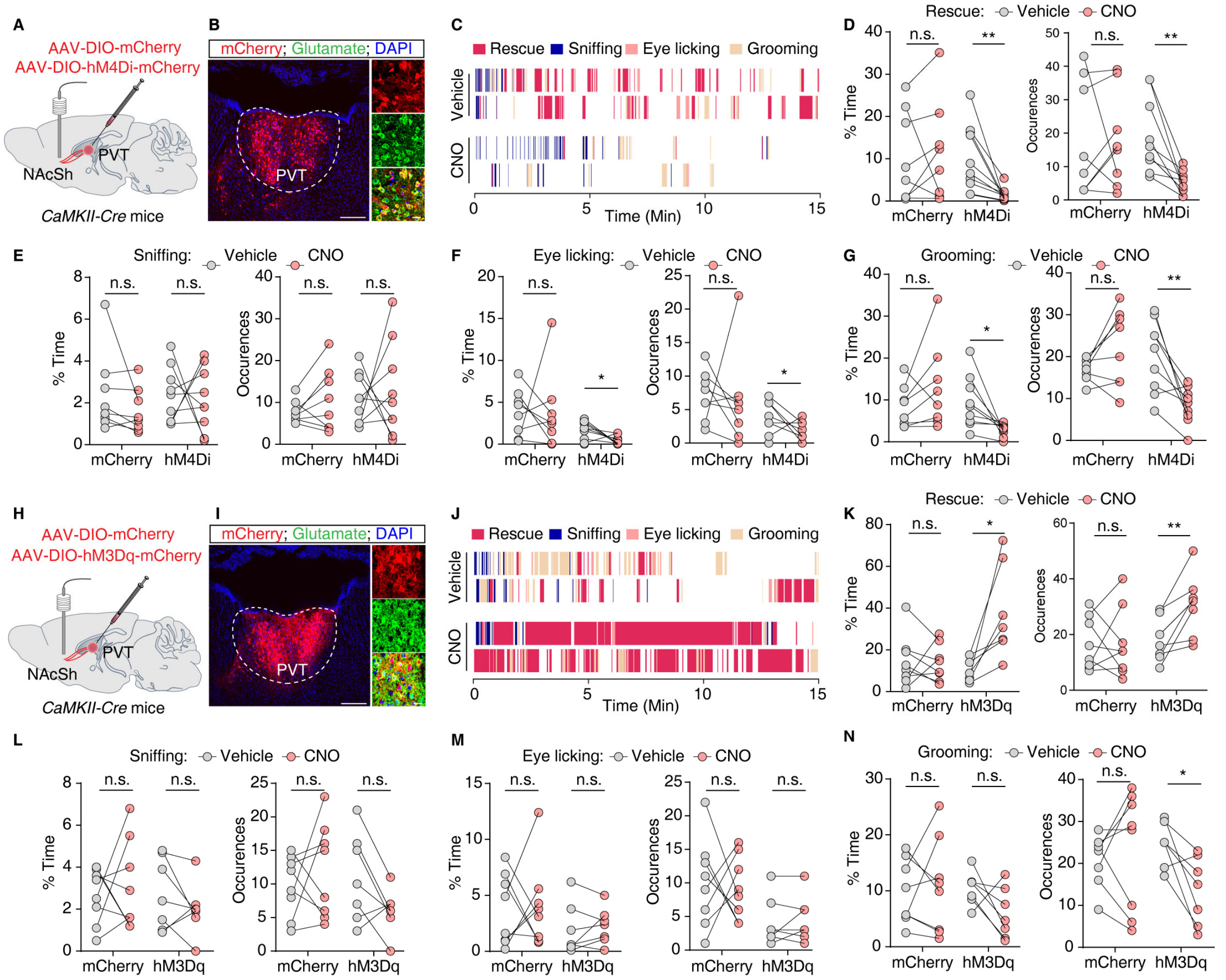

Fig. S14. PVT-NAcSh projections control rescue-like behavior. (**A**) Schematic for chemogenetic inhibition of the PVT^{Glu}→NAcSh circuit. (**B**) Representative images validate mCherry expression in PVT^{Glu} neurons of *CaMKII-Cre* mice. Scale bars, 200 µm (overview) and 20 µm (inset). (**C**) Example raster plots showing hM4Di-expressing bystander mice engage in rescue-like behavior, sniffing, eye licking, and grooming after treatment with vehicle or CNO. (**D**) Total duration percentage (left) and the number of occurrences (right) of rescue-like behavior in mCherry- and hM4Di-expressing bystander mice after treatment with vehicle or CNO. N (mCherry) = 8 mice; N (hM4Di) = 9 mice. (**E** to **G**) As indicated in panel D, total duration percentage and the number of occurrences for sniffing, eye licking, and grooming in mCherry- and hM4Di-expressing bystander mice, respectively. N (mCherry) = 8 mice; N (hM4Di) = 9 mice. (**H** and **I**) Schematic for chemogenetic activation of the PVT^{Glu}→NAcSh circuit. Scale bars, 200 µm (overview) and 20 µm (inset). (**J**) As indicated in panel C, example raster plots showing hM3Dq-expressing bystander

mice engage in various behaviors after treatment with vehicle or CNO. (**K**) As indicated in panel D, the data of recuse-like behavior in mCherry- and hM3Dq-expressing bystander mice. N (mCherry) = 8 mice; N (hM3Dq) = 7 mice. (**L** to **N**) As indicated in panel D, total duration percentage and the number of occurrences for sniffing, eye licking, and grooming in mCherry- and hM3Dq-expressing bystander mice, respectively. N (mCherry) = 8 mice; N (hM3Dq) = 7 mice. Data are presented as the mean ± SEMs. * $P < 0.05$, ** $P < 0.01$; n.s., not significant. Details of the statistical analyses are presented in Table S1.

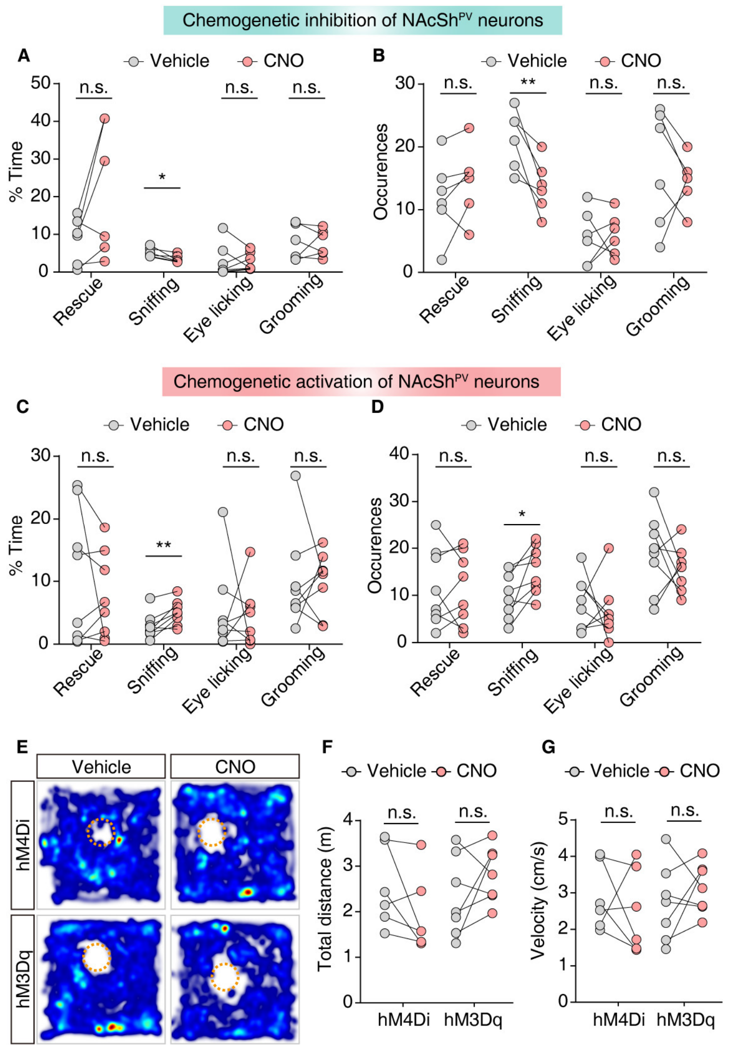

Fig. S15. Chemogenetic manipulation of NAcSh^{PV} neurons. (**A** and **B**) Total duration percentage (A) and the number of occurrences (B) for rescue-like behavior, sniffing, eye licking, and grooming in hM4Di-expressing bystander mice after treatment with vehicle or CNO. N = 6 mice. (**C** and **D**) Total duration percentage (C) and the number of occurrences (D) for rescue-like behavior, sniffing, eye licking, and grooming in hM3Dq-expressing bystander mice after treatment with vehicle or CNO. N = 8 mice. (**E**) Representative heatmaps showing the locations of hM4Di- or hM3Dq-expressing bystander mice paired with anesthetized mice (yellow dotted circle) in the open field test (OFT). (**F** and **G**) Summary data for total distance (F) and velocity (G) in the OFT from the indicated groups. N (hM4Di) = 6 mice; N (hM3Dq) = 7 mice. Data are presented as the mean ± SEMs. * $P < 0.05$, ** $P < 0.01$; n.s., not significant. Details of the statistical analyses are presented in Table S1.

Table S1. Statistical analyses related to Figures 1-6 and figures S1-15.

Fig.	Conditions (sample size)		Analysis	*P* value	t or F value
1C	AM: Empty (12)	AM: Subject (12)	Mann-Whitney test	$P <$ 0.0001	
	Toy: Empty (9)	Toy: Subject (9)	Mann-Whitney test	$P =$ 0.2581	
1D	AM: Empty (12)	AM: Subject (12)	Unpaired two sample *t* test	$P =$ 0.3685	t (22) = 0.9181
	Toy: Empty (9)	Toy: Subject (9)	Unpaired two sample *t* test	$P =$ 0.7024	t (16) = 0.3890
1E	AM: Empty (12)	AM: Subject (12)	Unpaired two sample *t* test	$P =$ 0.4485	t (22) = 0.7717
	Toy: Empty (9)	Toy: Subject (9)	Mann-Whitney test	$P =$ 0.2581	
1H	Tongue dragging: Control (10)	Tongue dragging: AM (10)	Mann-Whitney test	$P <$ 0.0001	
	Sniffing: Control (10)	Sniffing: AM (10)	Mann-Whitney test	$P =$ 0.0010	
	Eye licking: Control (10)	Eye licking: AM (10)	Mann-Whitney test	$P =$ 0.0325	
	Grooming: Control (10)	Grooming: AM (10)	Mann-Whitney test	$P =$ 0.0007	
1I	Tongue dragging: Control (10)	Tongue dragging: AM (10)	Mann-Whitney test	$P <$ 0.0001	
	Sniffing: Control (10)	Sniffing: AM (10)	Mann-Whitney test	$P =$ 0.0030	
	Eye licking: Control (10)	Eye licking: AM (10)	Mann-Whitney test	$P =$ 0.0325	
	Grooming: Control (10)	Grooming: AM (10)	Mann-Whitney test	$P =$ 0.0007	
1L (left)	Baseline (12), Grooming (19), Eye licking (14), Tongue dragging (21)		Kruskal-Wallis test	$P <$ 0.0001	
	Baseline (12)	Grooming (19)		$P =$ 0.0340	
	Baseline (12)	Eye licking (14)		$P =$ 0.0731	
	Baseline (12)	Tongue dragging (21)		$P <$ 0.0001	
	Grooming (19)	Tongue dragging (21)		$P =$ 0.0220	
	Eye licking (14)	Tongue dragging (21)		$P =$ 0.0342	
1L (right)	Baseline (12), Grooming (19), Eye licking (14), Tongue dragging (21)		Kruskal-Wallis test	$P <$0.0001	
	Baseline (12)	Grooming (19)		$P =$ 0.0027	

	Baseline (12)	Eye licking (14)		*P* = 0.0209	
	Baseline (12)	Tongue dragging (21)		*P* < 0.0001	
	Grooming (19)	Tongue dragging (21)		*P* = 0.0080	
	Eye licking (14)	Tongue dragging (21)		*P* = 0.0046	
1M	LORR: Control (9)	Rescued (10)	Unpaired two sample *t* test	*P* = 0.2748	t (17) = 1.128
	RORR: Control (9)	Rescued (10)	Unpaired two sample *t* test	*P* < 0.0001	t (17) = 5.008
1O	Control (10), Hindpaw (10), Back (10), Tongue (10)		Ordinary one-way ANOVA	*P* < 0.0001	F (3, 36) = 11.02
	Control (10)	Hindpaw (10)		*P* = 0.1874	
	Control (10)	Back (10)		*P* = 0.3307	
	Control (10)	Tingue (10)		*P* < 0.0001	
1Q	Wake: Control (9)	Wake: Pinch (9)	Mann-Whitney test	*P* = 0.5023	
	Maintenance: Control (9)	Maintenance: Pinch (9)	Unpaired two sample *t* test	*P* = 0.3106	t (16) = 1.047
	Emergence: Control (9)	Emergence: Pinch (9)	Unpaired two sample *t* test	*P* = 0.0195	t (16) = 2.597
3B	MTN: Control (8)	MTN: Rescue (8)	Mann-Whitney test	*P* = 0.0070	
	LC: Control (8)	LC: Rescue (8)	Unpaired two sample *t* test	*P* = 0.0005	t (14) = 4.542
3K	Control: Pre (9)	Control: Post (9)	Paired two sample *t* test	*P* = 0.0528	t (8) = 2.271
	Hindpaw: Pre (10)	Hindpaw: Post (10)	Paired two sample *t* test	*P* = 0.0266	t (9) = 2.648
	Back: Pre (10)	Back: Post (10)	Paired two sample *t* test	*P* = 0.0082	t (9) = 3.373
	Tongue: Pre (8)	Tongue: Post (8)	Wilcoxon matched-pairs signed rank test	*P* = 0.0078	
3M	Control (17), Hindpaw (17), Back (17), Tongue (18)		Kruskal-Wallis test	*P* = 0.0004	
	Control (17)	Hindpaw (17)		*P* > 0.9999	
	Control (17)	Back (17)		*P* > 0.9999	
	Control (17)	Tongue (18)		*P* = 0.0003	

4D	ACSF (5)	DNQX (5)	Paired two sample *t* test	*P* = 0.0006	t (4) = 9.870
4G	LORR: mCherry (8)	LORR: hM4Di (8)	Unpaired two sample *t* test	*P* = 0.0051	t (14) = 3.312
	RORR: mCherry ()	RORR: hM4Di ()	Unpaired two sample *t* test	*P* = 0.0135	t (14) = 2.825
4H	LORR: mCherry (10)	LORR: hM4Di (9)	Unpaired two sample *t* test	*P* = 0.8865	t (17) = 0.1448
	RORR: mCherry (10)	RORR: hM4Di (9)	Mann-Whitney test	*P* = 0.0001	
4J	mCherry (24)	hM4Di (23)	Mann-Whitney test	*P* < 0.0001	
4M	LORR: mCherry (8)	LORR: hM3Dq (8)	Unpaired two sample *t* test	*P* = 0.0053	t (14) = 3.297
	RORR: mCherry (8)	RORR: hM3Dq (8)	Unpaired two sample *t* test	*P* = 0.0485	t (14) = 2.161
4N	LORR: mCherry (8)	LORR: hM3Dq (8)	Unpaired two sample *t* test	*P* = 0.0219	t (14) = 2.578
	RORR: mCherry (8)	RORR: hM3Dq (8)	Unpaired two sample *t* test	*P* = 0.0007	t (14) = 4.300
4P	mCherry (24)	hM3Dq (25)	Unpaired two sample *t* test	*P* < 0.0001	t (47) = 5.330
4R	LORR: ACSF (9)	LORR: Caspase3 (9)	Unpaired two sample *t* test	*P* = 0.1828	t (16) = 1.393
	RORR: ACSF (9)	RORR: Caspase3 (9)	Unpaired two sample *t* test	*P* = 0.0372	t (16) = 2.273
5B	Sniffing: 410 (18)	Sniffing: 470 (18)	Wilcoxon matched-pairs signed rank test	*P* < 0.0001	
	Grooming: 410 (21)	Grooming: 470 (21)	Wilcoxon matched-pairs signed rank test	*P* = 0.0888	
	Eye licking: 410 (22)	Eye licking: 470 (22)	Wilcoxon matched-pairs signed rank test	*P* = 0.3359	
	Tongue dragging: 410 (33)	Tongue dragging: 470 (33)	Paired two sample *t* test	*P* < 0.0001	t (32) = 6.840
5E	Sniffing: 410 (26)	Sniffing: 470 (26)	Paired two sample *t* test	*P* < 0.0001	t (25) = 8.084
	Grooming: 410 (26)	Grooming: 470 (26)	Paired two sample *t* test	*P* < 0.0001	t (25) = 6.714

	Eye licking: 410 (24)	Eye licking: 470 (24)	Paired two sample *t* test	*P* = 0.7034	t (23) = 0.3856
	Tongue dragging: 410 (32)	Tongue dragging: 470 (32)	Wilcoxon matched-pairs signed rank test	*P* < 0.0001	
5J	Sniffing (62), Grooming (84), Eye licking (46), Rescue (76)		Kruskal-Wallis test	*P* < 0.0001	
	Sniffing (62)	Grooming (84)		*P* < 0.0001	
	Sniffing (62)	Eye licking (46)		*P* = 0.0048	
	Sniffing (62)	Rescue (76)		*P* < 0.0001	
5K	Sniffing (62), Grooming (84), Eye licking (46), Rescue (76)		Kruskal-Wallis test	*P* = 0.0017	
	Rescue (76)	Sniffing (62)		*P* = 0.0134	
	Rescue (76)	Grooming (84)		*P* = 0.0428	
	Rescue (76)	Eye licking (46)		*P* = 0.0009	
5Q	ACSF (6)	DNQX (6)	Paired two sample *t* test	*P* = 0.0006	t (5) = 7.686
5S	Vehicle (6)	CNO (6)	Paired two sample *t* test	*P* = 0.0267	t (5) = 3.104
5T	Vehicle (6)	CNO (6)	Wilcoxon matched-pairs signed rank test	*P* = 0.0312	
5V	Vehicle (8)	CNO (8)	Paired two sample *t* test	*P* = 0.0056	t (7) = 3.936
5W	Vehicle (8)	CNO (8)	Wilcoxon matched-pairs signed rank test	*P* = 0.0078	
6C	Sniffing: 410 (18)	Sniffing: 470 (18)	Paired two sample *t* test	*P* < 0.0001	t (17) = 9.640
	Grooming: 410 (18)	Grooming: 470 (18)	Paired two sample *t* test	*P* = 0.8419	t (17) = 0.2025
	Eye licking: 410 (8)	Eye licking: 470 (8)	Paired two sample *t* test	*P* = 0.5526	t (7) = 0.6237
	Tongue dragging: 410 (23)	Tongue dragging: 470 (23)	Wilcoxon matched-pairs signed rank test	*P* < 0.0001	

6E	Sniffing: 410 (32)	Sniffing: 470 (32)	Paired two sample *t* test	*P* = 0.0275	t (31) = 2.313
	Grooming: 410 (18)	Grooming: 470 (18)	Paired two sample *t* test	*P* =0.4043	t (17) = 0.8553
	Eye licking: 410 (23)	Eye licking: 470 (23)	Paired two sample *t* test	*P* = 0.8708	t (22) = 0.1646
	Tongue dragging: 410 (32)	Tongue dragging: 470 (32)	Paired two sample *t* test	*P* = 0.0084	t (31) = 2.816
6G	Vehicle (6)	CNO (6)	Wilcoxon matched-pairs signed rank test	*P* = 0.0312	
6H	Vehicle (6)	CNO (6)	Paired two sample *t* test	*P* = 0.0452	t (5) = 2.654
6J	Vehicle (6)	CNO (6)	Paired two sample *t* test	*P* = 0.0231	t (5) = 3.233
6K	Vehicle (6)	CNO (6)	Paired two sample *t* test	*P* = 0.0017	t (5) = 6.119
6M	Vehicle (6)	CNO (6)	Wilcoxon matched-pairs signed rank test	*P* = 0.6250	
6N	Vehicle (6)	CNO (6)	Paired two sample *t* test	*P* = 0.1711	t (5) = 1.597
6P	Vehicle (6)	CNO (6)	Paired two sample *t* test	*P* = 0.2460	t (5) = 1.314
6Q	Vehicle (6)	CNO (6)	Paired two sample *t* test	*P* = 0.2149	t (5) = 1.420
S1C	Tongue dragging: Cagemate (12)	Tongue dragging: Stranger (15)	Mann-Whitney test	*P* = 0.0725	
	Sniffing: Cagemate (12)	Sniffing: Stranger (15)	Mann-Whitney test	*P* = 0.9522	
	Eye licking: Cagemate (12)	Eye licking: Stranger (15)	Mann-Whitney test	*P* = 0.6220	
	Grooming: Cagemate (12)	Grooming: Stranger (15)	Unpaired two sample *t* test	*P* = 0.2966	t (25) = 1.066
S1D	Tongue dragging: Cagemate (12)	Tongue dragging: Stranger (15)	Mann-Whitney test	*P* = 0.1479	
	Sniffing: Cagemate (12)	Sniffing: Stranger (15)	Unpaired two sample *t* test	*P* = 0.1903	t (25) = 1.346
	Eye licking: Cagemate (12)	Eye licking: Stranger (15)	Unpaired two sample *t* test	*P* = 0.9525	t (25) = 0.0601

	Grooming: Cagemate (12)	Grooming: Stranger (15)	Mann-Whitney test	*P* = 0.2599	
S2B	Tongue dragging: Control (7)	Tongue dragging: AM (9)	Mann-Whitney test	*P* = 0.0002	
	Sniffing: Control (7)	Sniffing: AM (9)	Unpaired two sample *t* test	*P* < 0.0001	t (14) = 7.662
	Eye licking: Control (7)	Eye licking: AM (9)	Mann-Whitney test	*P* = 0.0002	
	Grooming: Control (7)	Grooming: AM (9)	Mann-Whitney test	*P* = 0.0002	
S2C	Tongue dragging: Control (7)	Tongue dragging: AM (9)	Mann-Whitney test	*P* = 0.0002	
	Sniffing: Control (7)	Sniffing: AM (9)	Unpaired two sample *t* test	*P* < 0.0001	t (14) = 7.836
	Eye licking: Control (7)	Eye licking: AM (9)	Mann-Whitney test	*P* < 0.0001	
	Grooming: Control (7)	Grooming: AM (9)	Unpaired two sample *t* test	*P* < 0.0001	t (14) = 7.344
S2F	Tongue dragging: Cagemate (10)	Tongue dragging: Stranger (10)	Mann-Whitney test	*P* = 0.0121	
	Sniffing: Cagemate (10)	Sniffing: Stranger (10)	Mann-Whitney test	*P* = 0.1375	
	Eye licking: Cagemate (10)	Eye licking: Stranger (10)	Unpaired two sample *t* test	*P* = 0.8822	t (18) =0.1503
	Grooming: Cagemate (10)	Grooming: Stranger (10)	Unpaired two sample *t* test	*P* = 0.5078	t (18) = 0.6758
S2G	Tongue dragging: Cagemate (10)	Tongue dragging: Stranger (10)	Unpaired two sample *t* test	*P* = 0.0048	t (18) = 3.217
	Sniffing: Cagemate (10)	Sniffing: Stranger (10)	Mann-Whitney test	*P* = 0.1653	
	Eye licking: Cagemate (10)	Eye licking: Stranger (10)	Unpaired two sample *t* test	*P* = 0.5544	t (18) 0.6024
	Grooming: Cagemate (10)	Grooming: Stranger (10)	Unpaired two sample *t* test	*P* = 0.3954	t (18) = 0.8707
S3B	AM (7)	Cadaver (7)	Mann-Whitney test	*P* = 0.3829	
S3C	AM (7)	Cadaver (7)	Unpaired two sample *t* test	*P* = 0.5606	t (12) = 0.5985

S3D	AM (7)	Cadaver (7)	Unpaired two sample *t* test	*P* = 0.0886	
S3F	Tongue dragging: AM (7)	Tongue dragging: Cavader (7)	Unpaired two sample *t* test	*P* = 0.0145	t (12) = 2.856
	Sniffing: AM (7)	Sniffing: Cavader (7)	Unpaired two sample *t* test	*P* = 0.0758	t (12) = 1.943
	Eye licking: AM (7)	Eye licking: Cavader (7)	Mann-Whitney test	*P* =0.0379	
	Grooming: AM (7)	Grooming: Cavader (7)	Unpaired two sample *t* test	*P* = 0.2076	t (12) = 1.332
S3G	Tongue dragging: AM (7)	Tongue dragging: Cavader (7)	Unpaired two sample *t* test	*P* = 0.0045	t (12) = 3.489
	Sniffing: AM (7)	Sniffing: Cavader (7)	Unpaired two sample *t* test	*P* = 0.0101	t (12) = 3.051
	Eye licking: AM (7)	Eye licking: Cavader (7)	Unpaired two sample *t* test	*P* = 0.0445	t (12) 2.243
	Grooming: AM (7)	Grooming: Cavader (7)	Mann-Whitney test	*P* = 0.2769	
S4A	Control (10)	Hindpaw (10)	Unpaired two sample *t* test	*P* = 0.7874	t (18) = 0.2738
	Control (10)	Back (10)	Unpaired two sample *t* test	*P* = 0.9663	t (18) = 0.0428
	Control (10)	Tongue (10)	Unpaired two sample *t* test	*P* = 0.3996	t (18) = 0.8629
S4C	Control (12)	Hindpaw (12)	Unpaired two sample *t* test	*P* = 0.6302	t (22) = 0.4882
	Control (12)	Back (12)	Unpaired two sample *t* test	*P* = 0.5777	t (22) = 0.5652
	Control (12)	Tongue (12)	Unpaired two sample *t* test	*P* = 0.9829	t (22) = 0.0217
S4D	Control (12)	Hindpaw (12)	Unpaired two sample *t* test	*P* = 0.8180	t (22) = 0.2329
	Control (12)	Back (12)	Unpaired two sample *t* test	*P* = 0.9377	t (22) = 0.0791
	Control (12)	Tongue (12)	Unpaired two sample *t* test	*P* = 0.0002	t (22) = 4.465
S4F	Control (7), Anesthesia (7), Pinch (7)		Ordinary one-way ANOVA	*P* < 0.0001	F (2, 18) = 34.86
	Control (7)	Anesthesia (7)		*P* < 0.0001	

	Control (7)	Pinch (7)		*P* = 0.0243	
S8F	Control (18), Hindpaw (16), Back (16), Tongue (19)		Ordinary one-way ANOVA	*P* = 0.0496	F (3, 65) = 2.752
	Control (18)	Hindpaw (16)		*P* = 0.9566	
	Control (18)	Back (16)		*P* = 0.7497	
	Control (18)	Tongue (19)		*P* = 0.3309	
S8H	Control (17), Hindpaw (17), Back (16), Tongue (17)		Ordinary one-way ANOVA	*P* = 0.4397	F (3, 63) = 0.9134
	Control (17)	Hindpaw (17)		*P* = 0.6507	
	Control (17)	Back (16)		*P* = 0.7554	
	Control (17)	Tongue (17)		*P* = 0.3877	
S9C	mCherry (4)	hM4Di (4)	Two-way RM ANOVA with Bonferroni post hoc analysis	*P* < 0.0001	F (1, 6) = 178.6
S9F	mCherry (4)	hM3Dq (4)	Two-way RM ANOVA with Bonferroni post hoc analysis	*P* = 0.0008	F (1, 6) = 38.00
S9I	ACSF (5)	Caspase3 (5)	Mann-Whitney test	*P* = 0.0079	
S10C	mPFC: Control (6)	mPFC: Rescue (6)	Unpaired two sample *t* test	*P* = 0.8802	t (10) = 0.1547
	ACC: Control (6)	ACC: Rescue (6)	Unpaired two sample *t* test	*P* = 0.1290	t (10) = 1.655
	LS: Control (6)	LS: Rescue (6)	Unpaired two sample *t* test	*P* = 0.1472	t (10) = 1.571
	CL: Control (6)	CL: Rescue (6)	Unpaired two sample *t* test	*P* = 0.8864	t (10) = 0.1465
	DEn: Control (6)	DEn: Rescue (6)	Unpaired two sample *t* test	*P* = 0.6129	t (10) = 0.5222
	NAc: Control (6)	NAc: Rescue (6)	Unpaired two sample *t* test	*P* = 0.0044	t (10) = 3.664
	BNST: Control (6)	BNST: Rescue (6)	Unpaired two sample *t* test	*P* = 0.4930	t (10) = 0.7116
	mPOA: Control (6)	mPOA: Rescue (6)	Unpaired two sample *t* test	*P* = 0.4105	t (10) = 0.8589
	PVT: Control (6)	PVT: Rescue (6)	Unpaired two sample *t* test	*P* = 0.0011	t (10) = 4.540
	PVN: Control (6)	PVN: Rescue (6)	Unpaired two sample *t* test	*P* = 0.8284	t (10) = 0.2225
	AHN: Control (6)	AHN: Rescue (6)	Mann-Whitney test	*P* = 0.0065	

	SCN: Control (6)	SCN: Rescue (6)	Mann-Whitney test	*P* = 0.0130	
	BLA: Control (6)	BLA: Rescue (6)	Unpaired two sample *t* test	*P* > 0.9999	t (10) = 0.000
	CeA: Control (6)	CeA: Rescue (6)	Unpaired two sample *t* test	*P* = 0.3229	t (10) = 1.040
	HIP: Control (6)	HIP: Rescue (6)	Unpaired two sample *t* test	*P* = 0.7560	t (10) = 0.3194
	vLGN: Control (6)	vLGN: Rescue (6)	Unpaired two sample *t* test	*P* = 0.3363	t (10) = 1.010
	VTA: Control (6)	VTA: Rescue (6)	Unpaired two sample *t* test	*P* = 0.6619	t (10) = 0.4506
	VC: Control (6)	VC: Rescue (6)	Unpaired two sample *t* test	*P* = 0.7795	t (10) = 0.2876
	DRN: Control (6)	DRN: Rescue (6)	Unpaired two sample *t* test	*P* = 0.5995	t (10) = 0.5423
	SC: Control (6)	SC: Rescue (6)	Unpaired two sample *t* test	*P* = 0.4450	t (10) = 0.7951
	PAG: Control (6)	PAG: Rescue (6)	Unpaired two sample *t* test	*P* = 0.7939	t (10) = 0.2683
S11B	Control (6)	Rescue (6)	Unpaired two sample *t* test	*P* = 0.0011	t (10) = 4.540
S11D	Control (6)	Rescue (6)	Unpaired two sample *t* test	*P* = 0.0044	t (10) = 3.664
S14D	%Time of mCherry: Vehicle (8)	%Time of mCherry: CNO (8)	Paired two sample *t* test	*P* = 0.7400	t (7) = 0.3453
	%Time of hM4Di: Vehicle (9)	%Time of hM4Di: CNO (9)	Wilcoxon matched-pairs signed rank test	*P* = 0.0039	
	Occurrences of mCherry: Vehicle (8)	Occurrences of mCherry: CNO (8)	Wilcoxon matched-pairs signed rank test	*P* = 0.5859	
	Occurrences of hM4Di: Vehicle (9)	Occurrences of hM4Di: CNO (9)	Paired two sample *t* test	*P* = 0.0039	t (8) = 4.006
S14E	%Time of mCherry: Vehicle (8)	%Time of mCherry: CNO (8)	Wilcoxon matched-pairs signed rank test	*P* = 0.1484	
	%Time of hM4Di: Vehicle (9)	%Time of hM4Di: CNO (9)	Paired two sample *t* test	*P* = 0.8048	t (8) = 0.2555
	Occurrences of mCherry: Vehicle (8)	Occurrences of mCherry: CNO (8)	Paired two sample *t* test	*P* = 0.2277	t (7) = 1.322
	Occurrences of hM4Di: Vehicle (9)	Occurrences of hM4Di: CNO (9)	Paired two sample *t* test	*P* = 0.7299	t (8) = 0.3576

S14F	%Time of mCherry: Vehicle (8)	%Time of mCherry: CNO (8)	Wilcoxon matched-pairs signed rank test	*P* = 0.5469	
	%Time of hM4Di: Vehicle (9)	%Time of hM4Di: CNO (9)	Wilcoxon matched-pairs signed rank test	*P* = 0.0547	
	Occurrences of mCherry: Vehicle (8)	Occurrences of mCherry: CNO (8)	Wilcoxon matched-pairs signed rank test	*P* = 0.2578	
	Occurrences of hM4Di: Vehicle (9)	Occurrences of hM4Di: CNO (9)	Paired two sample *t* test	*P* = 0.0448	t (8) = 2.376
S14G	%Time of mCherry: Vehicle (8)	%Time of mCherry: CNO (8)	Paired two sample *t* test	*P* = 0.1697	t (7) = 1.531
	%Time of hM4Di: Vehicle (9)	%Time of hM4Di: CNO (9)	Paired two sample *t* test	*P* = 0.0181	t (8) = 2.963
	Occurrences of mCherry: Vehicle (8)	Occurrences of mCherry: CNO (8)	Paired two sample *t* test	*P* = 0.0997	t (7) = 1.897
	Occurrences of hM4Di: Vehicle (9)	Occurrences of hM4Di: CNO (9)	Paired two sample *t* test	*P* = 0.0044	t (8) = 3.927
S14K	%Time of mCherry: Vehicle (8)	%Time of mCherry: CNO (8)	Paired two sample *t* test	*P* = 0.8348	t (7) = 0.2164
	%Time of hM3Dq: Vehicle (7)	%Time of hM3Dq: CNO (7)	Paired two sample *t* test	*P* = 0.0169	t (6) = 3.278
	Occurrences of mCherry: Vehicle (8)	Occurrences of mCherry: CNO (8)	Paired two sample *t* test	*P* = 0.9589	t (7) = 0.0534
	Occurrences of hM3Dq: Vehicle (7)	Occurrences of hM3Dq: CNO (7)	Paired two sample *t* test	*P* = 0.0092	t (6) = 3.775
S14L	%Time of mCherry: Vehicle (8)	%Time of mCherry: CNO (8)	Paired two sample *t* test	*P* = 0.6507	t (7) = 0.4728
	%Time of hM3Dq: Vehicle (7)	%Time of hM3Dq: CNO (7)	Paired two sample *t* test	*P* = 0.2563	t (6) = 1.255
	Occurrences of mCherry: Vehicle (8)	Occurrences of mCherry: CNO (8)	Paired two sample *t* test	*P* = 0.5004	t (7) = 0.7105
	Occurrences of hM3Dq: Vehicle (7)	Occurrences of hM3Dq: CNO (7)	Paired two sample *t* test	*P* = 0.0667	t (6) = 2.236

S14M	%Time of mCherry: Vehicle (8)	%Time of mCherry: CNO (8)	Wilcoxon matched-pairs signed rank test	*P* = 0.9453	
	%Time of hM3Dq: Vehicle (7)	%Time of hM3Dq: CNO (7)	Paired two sample *t* test	*P* = 0.5747	t (6) = 0.5932
	Occurrences of mCherry: Vehicle (8)	Occurrences of mCherry: CNO (8)	Paired two sample *t* test	*P* = 0.8349	t (7) = 0.2163
	Occurrences of hM3Dq: Vehicle (7)	Occurrences of hM3Dq: CNO (7)	Paired two sample *t* test	*P* = 0.5729	t (6) = 0.5960
S14N	%Time of mCherry: Vehicle (8)	%Time of mCherry: CNO (8)	Paired two sample *t* test	*P* = 0.7209	t (7) = 0.3719
	%Time of hM3Dq: Vehicle (7)	%Time of hM3Dq: CNO (7)	Paired two sample *t* test	*P* = 0.0943	t (6) = 1.985
	Occurrences of mCherry: Vehicle (8)	Occurrences of mCherry: CNO (8)	Paired two sample *t* test	*P* = 0.6153	t (7) = 0.5257
	Occurrences of hM3Dq: Vehicle (7)	Occurrences of hM3Dq: CNO (7)	Paired two sample *t* test	*P* = 0.0168	t (8) = 3.281
S15A	Rescue: Vehicle (6)	Rescue: CNO (6)	Paired two sample *t* test	*P* = 0.0715	t (5) = 2.280
	Sniffing: Vehicle (6)	Sniffing: CNO (6)	Paired two sample *t* test	*P* = 0.0267	t (5) = 3.104
	Eye licking: Vehicle (6)	Eye licking: CNO (6)	Paired two sample *t* test	*P* = 0.7760	t (5) = 0.3004
	Grooming: Vehicle (6)	Grooming: CNO (6)	Paired two sample *t* test	*P* = 0.9365	t (5) = 0.0838
S15B	Rescue: Vehicle (6)	Rescue: CNO (6)	Paired two sample *t* test	*P* = 0.2159	t (5) = 1.416
	Sniffing: Vehicle (6)	Sniffing: CNO (6)	Paired two sample *t* test	*P* = 0.0094	t (5) = 4.092
	Eye licking: Vehicle (6)	Eye licking: CNO (6)	Paired two sample *t* test	*P* = 0.8682	t (5) = 0.1746
	Grooming: Vehicle (6)	Grooming: CNO (6)	Paired two sample *t* test	*P* = 0.5475	t (5) = 0.6447
S15C	Rescue: Vehicle (8)	Rescue: CNO (8)	Paired two sample *t* test	*P* = 0.3655	t (7) = 0.9674
	Sniffing: Vehicle (8)	Sniffing: CNO (8)	Paired two sample *t* test	*P* = 0.0056	t (7) = 3.936

	Eye licking: Vehicle (8)	Eye licking: CNO (8)	Wilcoxon matched-pairs signed rank test	*P* = 0.9453	
	Grooming: Vehicle (8)	Grooming: CNO (8)	Wilcoxon matched-pairs signed rank test	*P* = 0.8672	
S15D	Rescue: Vehicle (8)	Rescue: CNO (8)	Paired two sample *t* test	*P* = 0.9068	t (7) = 0.1214
	Sniffing: Vehicle (8)	Sniffing: CNO (8)	Paired two sample *t* test	*P* = 0.0283	t (7) = 2.754
	Eye licking: Vehicle (8)	Eye licking: CNO (8)	Paired two sample *t* test	*P* = 0.6302	t (7) = 0.5033
	Grooming: Vehicle (8)	Grooming: CNO (8)	Paired two sample *t* test	*P* = 0.3766	t (7) = 0.9440
S15F	hM4Di: Vehicle (6)	hM4Di: CNO (6)	Paired two sample *t* test	*P* = 0.1576	t (5) = 1.661
	hM3Dq: Vehicle (7)	hM3Dq: CNO (7)	Paired two sample *t* test	*P* = 0.2050	t (6) = 1.421
S15G	hM4Di: Vehicle (6)	hM4Di: CNO (6)	Paired two sample *t* test	*P* = 0.5328	t (5) = 0.6696
	hM3Dq: Vehicle (7)	hM3Dq: CNO (7)	Paired two sample *t* test	*P* = 0.3865	t (6) = 0.9338

Table S2. Key resource table.

EAGENT or RESOURCE	SOURCE	IDENTIFIER
Antibodies		
Rabbit anti-Glutamate	Sigma-Aldrich	Cat# G6642; RRID: AB_259946
Rabbit anti-GABA	Sigma-Aldrich	Cat# A2052; RRID: AB_477652
Rabbit anti-TH	Proteintech	Cat# 25859-1-AP; RRID: AB_2716568
Rabbit anti-c-Fos	Synaptic Systems	Cat# 226008; RRID： AB_2891278
Guinea pig anti-c-Fos	Synaptic Systems	Cat# 226308; RRID： AB_2905595
Rabbit anti-TUBB3	Sigma-Aldrich	Cat# T2200; RRID： AB_262133
Rabbit anti-PV	Abcam	Cat# ab11427; RRID： AB_298032
Mouse anti-SST	Santa Cruz Biotechnology	Cat# sc-74556; RRID： AB_2271061
Rabbit anti-CGRP	Sigma-Aldrich	Cat# C8198; RRID: AB_259091
Alexa fluor 488-anti-Rabbit secondary antibody	Invitrogen	Cat# A21206; RRID: AB_2535792
Alexa fluor 594-anti-Rabbit secondary antibody	Invitrogen	Cat# A21207; RRID: AB_141637
Alexa fluor 647-anti-Rabbit secondary antibody	Invitrogen	Cat# A31573; RRID: AB_2536183
Alexa fluor 488-anti-Guinea pig secondary antibody	Invitrogen	Cat# A11073; RRID: AB_2534117
Alexa fluor 488-anti-Mouse secondary antibody	Invitrogen	Cat# A21202; RRID: AB_141607
Bacterial and virus strains		
rAAV-CAG-DIO-EGFP-WPRE-hGH pA	BrainVTA	Cat# PT-0168
rAAV2/9-EF1α-DIO-mCherry-WPRE-hGH pA	BrainVTA	Cat# PT-0013
rAAV-EF1α-DIO-hChR2(H134R)-mCherry-WPRE-hGH pA	BrainVTA	Cat# PT-0002
ScAAV2/1-hSyn-Cre-WPRE-hGH pA	BrainVTA	Cat# PT-2334
rAAV2/1-hSyn-Cre-EGFP-WPRE-hGH pA	BrainVTA	Cat# PT-1168
rAAV2/Retro-hSyn-Cre-WPRE-hGH pA	BrainVTA	Cat# PT-0136
rAAV2/9-EF1α-DIO-hM3D(Gq)-mCherry	BrainCase	Cat# BC-0145
rAAV2/9-EF1α-DIO-hM4D(Gi)-EYFP	BrainCase	Cat# BC-0154
rAAV2/Retro-hSyn-EGFP-WPRE-hGH polyA	BrainVTA	Cat# PT-1990

AAV2/9-hSyn-DIO-jGCaMP7s-WPRE-pA	TailTool	Cat# S0590-9
rAAV2/9-nEF1α-fDIO-taCasp3-TEVp-WPRE-hGH pA	BrainVTA	Cat# PT-1383
rAAV2/Retro-hSyn-FLP-WPRE-hGH pA	BrainVTA	Cat# PT-0341
rAAV2/8-EF1α-DIO-oRVG-WPRE-hGH pA	BrainVTA	Cat# PT-0023
rAAV2/8-EF1α-DIO-mCherry-F2A-TVA-WPRE-hGH pA	BrainVTA	Cat# PT-0207
rAAV-CAG-DIO-mcherry-mcherry-WPREs	BrainVTA	Cat# PT-1529
PRV-CAG-EGFP	BrainVTA	Cat# P03001
rAAV2/Retro-hSyn-FLP-WPRE-hGH pA	BrainVTA	Cat# PT-0341
rAAV2/9-nEF1α-fDIO-hChR2(H134R)-EGFP-WPRE-hGH polyA	BrainVTA	Cat# PT-1384
Chemicals, peptides, and recombinant proteins		
6,7-dinitroquinoxaline-2,3-dione (DNQX)	Sigma-Aldrich	Cat# D0540
Clozapine-N-oxide (CNO)	MedChemExpress	Cat# HY-17366
Carprofen	Sigma-Aldrich	Cat# PHR1452
Dexamethasone	MedChemExpress	Cat# HY-14686
Enrofloxacin	MedChemExpress	Cat# HY-B0502
Experimental models: Organisms/strains		
Mouse: wild type C57BL/6J	Charles River	N/A
Mouse: *CaMKII-Cre*	Jackson Laboratory	005359; RRID: IMSR_JAX: 005359
Mouse: *VGluT2-Cre*	Jackson Laboratory	016963; RRID: IMSR_JAX:016963
Mouse: *PV-Cre*	Jackson Laboratory	008069; RRID: IMSR_JAX:008069
Mouse: *DBH-Cre*	Jackson Laboratory	033951; RRID: IMSR_JAX: 033951
Mouse: *D1-Cre*	Gensat	B6.FVB(Cg)-Tg(Drd1-cre)EY262Gsat/Mmucd
Mouse: *D2-Cre*	Gensat	B6.FVB(Cg)-Tg(Drd2-cre)ER44Gsat/Mmcd
Software and algorithms		
OriginPro 2017	Origin Lab	https://www.originlab.com/index.aspx?go=Products/Origin
Illustrator CS6	Adobe	https://www.adobe.com/products/illustrator.html
GraphPad Prism 8	GraphPad Software	https://www.graphpad.com/features
ZEN	Zeiss	https://www.zeiss.com/microscopy/en/products/software/zeiss-zen.html

EthoVision XT 14	Noulds	https://www.noldus.com/ethovision-xt
Fiji	National Institutes of Health	https://fiji.sc/
ImageJ	National Institutes of Health	https://imagej.net/ij/download.html.
MATLAB 2019b	MathWorks	https://ww2.mathworks.cn/en/products/matlab.html
BORIS	Friard and Gamba	http://www.boris.unito.it
Clampfit	Molecular Devices	https://www.moleculardevices.com/products/axon-patch-clamp-system/acquisition-and-analysis-software/pclamp-software-suite
NeuroExplorer 5	Plexon Inc	https://plexon.com/neuroexplorer-v50-neural-data-analysis-software/
IMARIS 9.6.2	Bitplane	https://imaris.oxinst.com/packages
Other		
Optogenetics fibers	Inper	N/A
Cannula	RWD	N/A

Movie S1.

Prosocial interactions between a bystander mouse and an anesthetized mouse.

Movie S2.

A bystander mouse engaging in tongue dragging towards an anesthetized mouse.

Movie S3.

3D visualization of the tongue-projecting MTN^{Glu} neuronal terminals ($mCherry^{+}$) in the trigeminal ganglion.

Movie S4.

3D visualization of the mCherry-labeled MTN^{Glu} neuronal terminals in the tongue.

Movie S5.

Microendoscopic calcium imaging for GCaMP7s-expressing NAcSh neurons from a bystander mouse.
